# A Survey of Research Participants’ Privacy-Related Experiences and Willingness to Share Real-World Data with Researchers

**DOI:** 10.3390/jpm12111922

**Published:** 2022-11-17

**Authors:** Rachele M. Hendricks-Sturrup, Fang Zhang, Christine Y. Lu

**Affiliations:** 1 Department of Population Medicine, Harvard Pilgrim Health Care Institute and Harvard Medical School, Boston, MA 02115, USA; 2 Duke-Margolis Center for Health Policy, Washington, DC 20004, USA; 3 Department of Interdisciplinary Health Studies, Ohio University, Athens, OH 45701, USA

**Keywords:** privacy, real-world data, real-world evidence, precision medicine, personalized medicine, research

## Abstract

Background: Real-world data (RWD) privacy is an increasingly complex topic within the scope of personalized medicine, as it implicates several sources of data. Objective: To assess how privacy-related experiences, when adjusted for age and education level, may shape adult research participants’ willingness to share various sources of real-world data with researchers. Methods: An electronic survey was conducted in April 2021 among adults (≥18 years of age) registered in ResearchMatch, a national health research registry. Descriptive analyses were conducted to assess survey participant demographics. Logistic regression was conducted to assess the association between participants’ five distinct privacy-related experiences and their willingness to share each of the 19 data sources with researchers, adjusting for education level and age range. Results: A total of 598 ResearchMatch adults were contacted and 402 completed the survey. Most respondents were over the age of 51 years (49% total) and held a master’s or bachelor’s degree (63% total). Over half of participants (54%) had their account accessed by someone without their permission. Almost half of participants (49%) reported the privacy of their personal information being violated. Analyses showed that, when adjusted for age range and education level, participants whose reputations were negatively affected as a result of information posted online were more likely to share electronic medical record data (OR = 2.074, 95% CI: 0.986–4.364) and genetic data (OR = 2.302, 95% CI: 0.894–5.93) versus those without this experience. Among participants who had an unpleasant experience as a result of giving out information online, those with some college/associates/trade school compared to those with a doctoral or other terminal degree were significantly more willing to share genetic data (OR = 1.064, 95% CI: 0.396–2.857). Across all privacy-related experiences, participants aged 18 to 30 were significantly more likely than those over 60 years to share music streaming data, ridesharing history data, and voting history data. Additionally, across all privacy-related experiences, those with a high school education were significantly more likely than those with a doctorate or other terminal degree to share credit card statement data. Conclusions: This study offers the first insights into how privacy-related experiences, adjusted for age range and education level, may shape ResearchMatch participants’ willingness to share several sources of real-world data sources with precision medicine researchers. Future work should further explore these insights.

## 1. Introduction

Real-world data (RWD) generally encompasses “data relating to patient health status and/or the delivery of health care, routinely collected from a variety of sources” [1]. RWD sources include, but are not limited to, electronic health record (EHR) data, hospital or insurance company’s administrative and claims data, patient-generated data (e.g., data generated by in-home or self-monitoring devices such as wearables and fitness trackers), consumer-generated data, and laboratory data. The definition of RWD has been conflated with the definition and uses of “big data” in health and/or medicine [2] This is particularly true in areas where enormous amounts of data are collected to drive artificial intelligence and machine learning (AI/ML) that may offer deeper insights into the patient journey, clinical and/or treatment outcomes among subpopulations and social determinants of health (SDOH).

**Figure 1:**
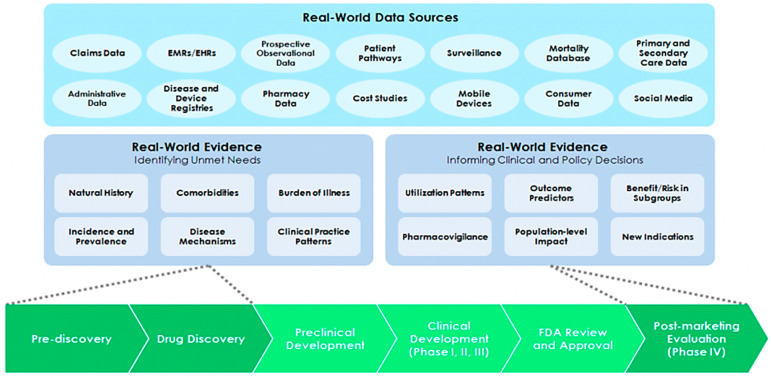
Summary of diverse real-world data sources and evidence used to inform drug discovery and clinical and policy decision-making (Reprinted/adapted with permission from Galson and Simon, 2016. National Academies Press) [3].

In their 2016 National Academy of Medicine discussion paper, Galson and Simon illustrated how insights from a variety of RWD sources can be leveraged to generate real-world evidence (RWE; see Figure 1) [3]. Importantly, such insights may identify unmet health care needs to drive drug discovery, as well as inform clinical and policy decisions to drive post-market evaluations of safety and impact across a range of new personalized medicines and medical devices. Building on this discussion is a recent study by Douglas and Kumar, which identified 72 precision medicine utilization studies published between 2015–2021 that incorporated one or more combinations of real-world data from various sources (administrative claims (commercial and public, 33% and 25%, respectively), clinical databases (29%), lab databases (31%), registries (55%), and integrated datasets from more than one source (38%)) [4].

Insurers or payers are also leveraging a variety of RWD sources today to build AI/ML-driven predictive models and risk scores that may identify members with or at risk of requiring complex, personalized health care needs, interventions, care coordination, and condition management [5]. Predictive modeling relies on a vast amount of RWD garnered from a variety of sources, including electronic health records, insurance claims, and genetic datasets, and may become augmented once combined with additional data reflecting the patient’s or patient populations’ natural and health system environments in which they live and/or navigate regularly (i.e., SDOH data reflecting health care access, healthy food access, poverty/wealth, education level, employment, housing, local or occupational hazard exposure, etc.). Predictive modeling also incorporates alternative data sources that can be used as a proxy for understanding a patient’s health status, such as voting registration data, where greater voting frequency can infer better health outcomes [6].

Overall, RWD sources offer powerful insights into patients’ health-related experiences, disease predispositions, and health beliefs. Yet, the current oversupply of and growing demand for RWD has led to a growing body of research that seeks to accomplish some of the earliest goals of personalized medicine: (1) “improve treatment outcomes and reduce adverse events that matter to both the clinician and patient” [7], (2) “to target the right treatments to the right patients at the right time” [8], and (3) “understand how a person’s genetics, environment, and lifestyle can help determine the best approach to prevent or treat disease” [9].

However, given the breadth and sensitivity of sources from which RWD may derive, RWD privacy and discretion is an increasingly complex and nebulous topic with unclear or unresolved concerns. Recent studies show that concerns may vary across a wide range of stakeholders, rendering their willingness to share or donate their RWD to health researchers likely subject to their unique data privacy experiences [10,11,12]. Complicating matters is the fact that consumer-generated data sources, including but not limited to social media or geolocation data, are generally considered non-health-related, yet can become health-related if the data are applied or interpreted in a manner of clinical or health relevance. This introduces unprecedented and unresolved ethical, legal, and social implications (ELSI) and considerations that may directly or indirectly influence individuals’ willingness to share their RWD with researchers. Indeed, policy, industry, academic, patient, and health system stakeholders have emphasized that “a critical challenge of our time is to embrace the enormous opportunity for using diverse sources of data to improve health while protecting the privacy of individuals” [13].

Amid mass commentary and discussion on ELSI considerations for consumer health data [10,11,14,15,16,17,18,19,20,21,22], and amid the increased online or digital engagement before and during the COVID-19 pandemic [23,24,25,26,27], few studies to date have assessed how privacy-related experiences among research participants might shape their willingness to share their real-world data with health researchers. This study seeks to fill this gap by exploring how five distinct privacy-related experiences, adjusted for age and education level, may shape adult research participants’ willingness to share 19 different real-world data sources.

## 2. Methods

### 2.1. Survey Development and Validation

An 18-item electronic survey was developed using Qualtrics software, where the survey contained four demographical items, three privacy threshold items, and two items focused on willingness to share data with researchers. Survey items published by Grande et al., Seltzer et al., and Zhu et al. were chosen, adapted, and re-validated among a convenience sample of five individuals who identify as both patients and health consumers, for bias, relevance, and cognition [10,28,29]. Based on the pilot participants’ feedback, the survey questions were refined to improve item quality and clarity, and overall instrument clarity, appropriateness, and relevance.

A recent systematic review and a survey study each determined that age, income level, and education level are the strongest predictors of online or digital footprint activity [30,31]. Therefore, we applied Zhu et al.’s demographic data collection convention; demographic data collected included age, education level, duration of using online medical websites (years), and annual frequency of getting ill [29]. The final survey consisted of four demographic items and 12 closed and two open-ended items focused on participants’ privacy concerns and perspectives (see full survey in Supplement).

### 2.2. Survey Population

ResearchMatch has been described as a “disease-neutral, Web-based recruitment registry to help match individuals who wish to participate in clinical research studies with researchers actively searching for volunteers throughout the US” [32]. Populations in ResearchMatch live within the US and Puerto Rico, are of all ages and races/ethnicities, and consist of healthy volunteers as well as those living with medical conditions. Individuals within the ResearchMatch database sign up to become volunteers through the ResearchMatch platform to support research studies. Access to the ResearchMatch platform for this study was provided through Ohio University. At the start of the survey, a total of 148,090 participants were registered in ResearchMatch.

### 2.3. Survey Distribution

In April 2021, the electronic survey was administered to adults (≥18 years of age) registered in the ResearchMatch database who agreed to be contacted to engage in the survey after receiving an informational electronic invitation letter via the ResearchMatch platform. As participants agreed to participate in the survey, they received an email correspondence with details about the study and a link to the electronic survey.

ResearchMatch participants were invited to complete the survey, regardless of health status, race, gender, or any other mutable or immutable characteristics. Participants’ personal contact details were received only after participants agreed to participate in the survey. Participant email addresses were deleted or destroyed to prevent reidentification at the conclusion of the study.

### 2.4. Survey Incentives and Completion Reminders

Participants who completed the survey held a random chance to receive a $250 (a total of two gift cards available), $100 (a total of four gift cards available), $50 (a total of six gift cards available), or $25 gift card (a total of 12 gift cards available). A random selection tool developed using Microsoft Excel was used to randomly select email addresses of survey participants and deliver the gift card incentives.

Survey participants were informed that their participation was entirely voluntary. No survey questions were mandatory, and participants were informed that they may skip any question(s) at any time. Survey participants were welcomed to contact the research team at any time with any questions or concerns about the study. Reminders were sent up to three times to participants who began but had yet to complete and submit the survey within the study timeframe.

### 2.5. Data Analysis

**Figure 2:**
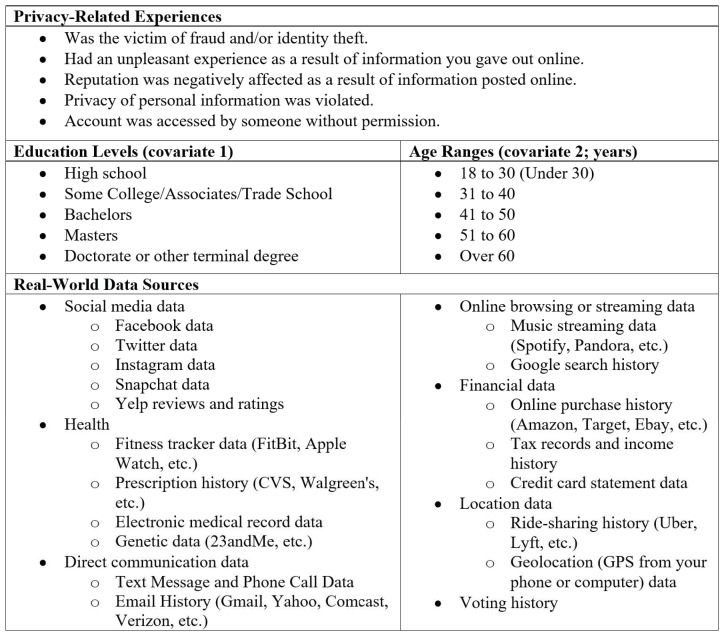
List of privacy-related experiences, age ranges, and education levels and real-world data sources.

The present analysis centers on ResearchMatch participants with a 100% response or completion rate (inclusive of completed surveys with items containing no responses). A Qualtrics software tool was used to calculate an ideal survey sample size (*n* = 384) based on the total ResearchMatch population (95% CI; 5% margin of error). Descriptive analyses were conducted using Microsoft Excel. Logistic regression was used to assess the association between participants’ five distinct privacy-related experiences and their willingness to share each of the 19 data sources with researchers (independent/outcome variables). Preliminary results suggest strong correlations between age and education level with respondents’ willingness to share data; thus, all logistic regressions were adjusted for education level (covariate 1; reference group = doctorate or other terminal degree) and age range (covariate 2; reference group = over 60 years; see Figure 2. Results were reported as adjusted odds ratios (ORs) with 95% confidence intervals. Results were considered statistically significant at *p* ≤ 0.05 and moderately significant at *p* ≤ 0.10 (although, for concision in our reporting, results discussed below are for findings at *p* ≤ 0.08). All logistic regression analyses were conducted using SAS version 9.4 (SAS Institute Inc., Cary, NC, USA).

### 2.6. Ethics Review, Oversight, and Approval

ResearchMatch is a registry and collaborative project that is maintained at Vanderbilt University and overseen by the Vanderbilt University Institutional Review Board. The present study was reviewed and approved by the Ohio University Institutional Review Board under protocol #20-E-457. ResearchMatch participants’ completion of the survey implied their consent to engage in the survey.

## 3. Results

### 3.1. Overall Assessment

A total of 598 volunteers agreed to receive direct invitations to participate in the survey. Following receipt of email invitations, 470 participants initiated the survey and 402 completed and submitted the survey (86% completion rate among those who initiated the survey). Three participants who initiated but did not complete and submit the survey cited reasons for their non-completion, which were that the participant either did not understand the way electronics affect health or did not understand the nature of the survey questions. One participant noted that their age (>55) could be a factor as to why the participant did not understand the nature of the survey questions.

### 3.2. Participant Characteristics

Table S1 (see Supplement) summarizes demographic characteristics among all survey participants/respondents who completed or submitted the survey. Most participants (49%) were over age 51. Most participants (87%) held either some college/associates/trade school, a bachelor’s degree, or master’s degree. Most participants (56%) reported using online medical websites for seven years or more. Most participants (94%) reported an annual frequency of illness of six occurrences or less.

### 3.3. Descriptive Overview of Past Personal Privacy-Related Experiences

**Figure 3:**
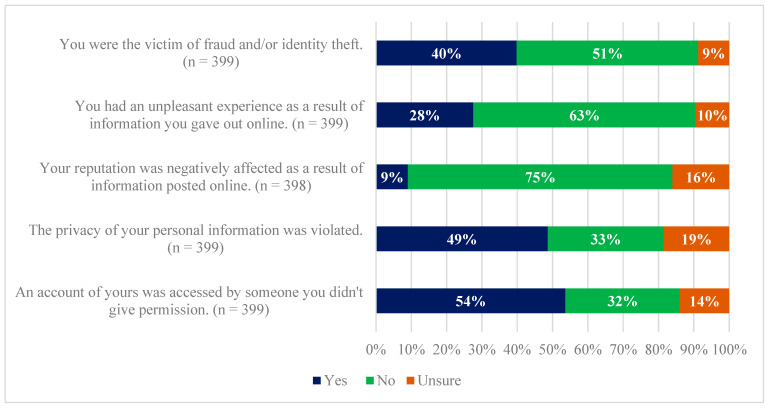
Reported past personal privacy-related experiences among survey participants.

Participants were asked if they had experienced one or more of five different privacy experiences or experiences (Figure 3). About half of participants (54%) had their account being accessed by someone without their permission. Nearly half of participants (49%) reported the privacy of their personal information being violated. Most participants (52% to 75%) reported never (1) being the victim of fraud and/or identity theft, (2) having an unpleasant experience following sharing information online, and (3) having their reputation negatively affected as a result of information posted online.

Table S2 summarizes participants’ willingness to donate data in the event they experienced being the victim of fraud and/or identity theft. Among participants who have been the victim of fraud and/or identify theft online (range between 155 to 158 responses), the greatest percent of respondents were willing to share prescription history data (50% of 157 responses) and unwilling to share tax records and income history (76% of 157 responses). Among participants who have not been the victim of fraud and/or identify theft online (range between 202 to 204 responses), the greatest percent of respondents were willing to share prescription history data (53% of 204 responses) and unwilling to share tax records and income history (76% of 204 responses).

Table S3 summarizes participants’ willingness to donate data in the event they had an unpleasant experience as a result of information given out online. Among participants who have had an unpleasant experience as a result of information given out online (range between 107 to 108 responses), the greatest percent of respondents were willing to share music streaming data (53% of 108 responses) and unwilling to share tax records and income history (78% of 108 responses). Among participants who have not had an unpleasant experience as a result of information given out online (range between 246 to 250 responses), the greatest percent of respondents were willing to share prescription history data (51% of 249 responses) and unwilling to share tax records and income history (77% of 249 responses).

Table S4 summarizes participants’ willingness to donate data in the event their reputations were negatively affected as a result of information posted online. Among participants who reported that their reputation was negatively affected as a result of information posted online (range between 34 to 35 responses), the greatest percent of respondents were willing to share online purchase history data (63% of 35 responses) and prescription history data (63% of 35 responses) and unwilling to share tax records and income history (77% of 35 responses). Among participants who reported that their reputation was not negatively affected as a result of information posted online (range between 293 to 296 responses), the greatest percent of respondents were willing to share prescription history data (50% of 295 responses) and unwilling to share tax records and income history (77% of 295 responses).

Table S5 summarizes participants’ willingness to donate data in the event they experienced a violation of personal information privacy. Among participants who experience a violation of personal information privacy (range between 193 to 194 responses), the greatest percent of respondents were willing to share fitness tracker data (48% of 194 responses) and unwilling to share tax records and income history (77% of 194 responses) and credit card statement data (77% of 193). Among participants who did not experience a violation of personal information privacy (range between 128 to 131 responses), the greatest percent of respondents were willing to share prescription history data (53% of 131 responses) and unwilling to share tax records and income history (78% of 130 responses).

Table S6 summarizes participants’ willingness to donate data in the event their accounts were accessed by someone without permission. Among participants who reported that their account was accessed by someone without permission (range between 191 to 193 responses), the greatest percent of respondents were willing to share music streaming data (49% of 193 responses and unwilling to share tax records and income history (75% of 192 responses). Among participants who reported that their account was not accessed by someone without permission (range between 126 to 128 responses), the greatest percent of respondents were willing to share voting history data (51% of 127 responses) and unwilling to share tax records and income history data (77% of 128 responses) and credit card statement data (77% of 128 responses).

### 3.4. Victim of Fraud and/or Identify Theft and Willingness to Donate Data

Within this group, adjusted logistical regression analyses suggest that participants who were victims of fraud and/or identify theft were less likely than those without this experience to share all forms of social media data with researchers, except Snapchat data (OR = 1.383, 95% CI: 0.57–3.354), though these findings were not significant (see Table S7). Modest associations were found among participants who were victims of fraud and/or identity theft and their willingness to share all other forms of real-world data with researchers, though these findings were also not significant.

Interestingly, however, within this group, logistical regression analyses suggest significant relationships between participants’ age ranges and their willingness to share prescription history data (18 to 30 years, OR = 1.323, 95% CI: 0.674–2.594); 41 to 50 years, OR = 0.518, 95% CI: 0.26–1.034), music streaming data (18 to 30 years, OR = 7.593, 95% CI: 3.026–19.051), tax records and income history data (31 to 40 years, OR = 1.747, 95% CI: 0.85–3.592), and voting history data (18 to 30 years, OR = 3.344, 95% CI: 1.661–6.729). Overall, participants under 40 years versus those over 60 years were significantly more likely to share these data sources. Yet, participants aged 41 to 50 years were significantly less likely than those over 60 years to share prescription history data with researchers.

Additionally, within this group, adjusted logistical regression analyses also suggest significant relationships between participants’ education levels and their willingness to share Snapchat data (some college/associates/trade school, OR = 0.2, 95% CI: 0.034–1.186), history data (masters, OR = 0.785, 95% CI: 0.34–1.813), text message and phone data (masters, OR = 0.528, 95% CI: 0.215–1.294), email history data (masters, OR = 0.676, 95% CI: 0.278–1.647), Google search history data (bachelors, OR = 0.659, 95% CI: 0.297–1.462; masters, OR = 0.601, 95% CI: 0.267–1.351), online purchase history data (high school, OR = 3.859, 95% CI: 0.701–21.242; masters, OR = 0.588, 95% CI: 0.258–1.339), credit card statement data (high school, OR = 3.504, 95% CI: 0.715–17.178; bachelors, OR = 0.72, 95% CI: 0.321–1.615; masters, OR = 0.621, 95% CI: 0.227–1.701), and geolocation data (masters, OR = 0.533, 95% CI: 0.229–1.237). Overall, and interestingly, participants with only high school education were significantly more likely than those with a doctorate or other terminal degree to share these data sources with researchers, yet participants with a master’s degree versus those with a doctorate or other terminal degree were less likely to share these data sources. Those with some college/associates/trade school education were also significantly less likely than those with a doctorate or other terminal degree to share Snapchat data.

### 3.5. Having an Unpleasant Experience as a Result of Information Given out Online and Willingness to Donate Data

Adjusted logistical regression analyses suggest modest yet insignificant relationships between participants having an unpleasant experience after giving out their information online and their willingness to share real-world data with researchers (versus those without this experience). Participants who had an unpleasant experience after giving out their information online were less likely to share Twitter data (OR = 0.729, 95% CI: 0.304–1.752) than those without this experience, though this result was not significant (see Table S8).

Within this group, adjusted logistical regression analyses suggest significant relationships between participants’ age ranges and their willingness to share Facebook data (51 to 60 years, OR = 2.044, 95% CI: 0.933–4.476), Twitter data (51 to 60 years, OR = 5.728, 95% CI: 1.443–22.735), prescription history data (41 to 50 years, OR = 0.5, 95% CI: 0.248–1.009), email history data (51 to 60 years, OR = 1.457, 95% CI: 0.759–2.796), music streaming data (18 to 30 years, OR = 6.702, 95% CI: 2.812–15.97), Google search history data (51 to 60 years, OR = 1.657, 95% CI: 0.885–3.103), and voting history data (18 to 30 years, OR = 3.023, 95% CI: 1.533–5.959). Interestingly, participants aged between 51 to 60 years were significantly more likely to share Twitter data than those over 60 years and participants aged between 41 to 50 years were significantly less likely than participants over 60 years to share prescription history data with researchers. Also interesting, participants aged under 30 years were significantly more likely than participants over 60 years to share music streaming data and voting history data with researchers. Lastly, participants aged 51 to 60 years were significantly more likely than those over 60 years to share Google search history data with researchers.

Within this same group, adjusted logistical regression analyses suggest significant relationships between participants’ education levels and their willingness to share Snapchat data (some college/associates/trade school, OR = 0.224, 95% CI: 0.037–1.353), genetic data (some college/associates/trade school, OR = 1.064, 95% CI: 0.396–2.857), email history data (master’s, OR = 0.801, 95% CI: 0.309–2.074), Google search history data (high school, OR = 3.227, 95% CI: 0.7–14.888; bachelor’s, OR = 0.722, 95% CI: 0.317–1.643; master’s, OR = 0.738, 95% CI: 0.321–1.697), online purchase history data (high school, OR = 8.378, 95% CI: 0.927–75.706; bachelor’s, OR = 0.791, CI: 0.345–1.811; master’s, OR = 0.696, 95% CI: 0.3–1.619), credit card statement data (high school, OR = 3.52, 95% CI: 0.7–17.706; master’s, OR = 0.704, 95% CI: 0.245–2.021), and geolocation data (master’s, OR = 0.601, 95% CI: 0.253–1.431). Overall, participants with a bachelor’s or master’s degree were significantly less likely than participants with a doctorate or other terminal degree to share email history data, Google search history data, online purchase history data, credit card statement data, and geolocation data with researchers. Respectively, participants with only high school education were significantly more likely than participants with a doctorate or other terminal degree to share those data sources with researchers. Lastly, participants with some college/associates/trade school education were significantly less likely than those with a doctorate or other terminal degree to share Snapchat data.

### 3.6. Reputation Being Negatively Affected as a Result of Information Posted Online and Willingness to Donate Data

Interestingly, adjusted logistical regression analyses suggest that participants whose reputations were negatively affected as a result of information posted online were modestly to more willing to share all sources of real-world data with researchers (ranging from voting history data, OR = 1.175, CI: 0.552–2.501 to Twitter data, OR = 3.278, CI: 0.608–17.678) than those who were not. Although, these results were significant only for electronic medical record data (OR = 2.074, 95% CI: 0.986–4.364), genetic data (OR = 2.302, 95% CI: 0.894–5.93), and music streaming data (OR = 2.851, 95% CI: 0.997–8.147; see Table S9).

Within this group, adjusted logistical regression analyses suggest significant relationships between participants’ age ranges and their willingness to share Facebook data (41 to 50 years, OR = 0.555, 95% CI: 0.239–1.288), prescription history data (41 to 50 years, OR = 0.436, 95% CI: 0.21–0.903), music streaming data (18 to 30 years, OR = 6.48, 95% CI: 2.607–16.102), tax records and income history data (31 to 40 years, OR = 1.749, 95% CI: 0.822–3.725), voting history data (18 to 30 years, OR = 2.835, 95% CI: 1.394–5.766) with researchers. Overall, participants aged 41 to 50 years are less willing to share Facebook data than participants over 60 years, yet participants aged 31 to 40 years were significantly more willing to share tax records and income history data with researchers.

Within this same group, adjusted logistical regression analyses also suggest significant relationships between participants’ education levels and their willingness to share text message and phone data (master’s, OR = 0.544, 95% CI: 0.216–1.37), email history data (high school, OR = 4.022, 95% CI: 0.893–18.118; master’s, OR = 0.732, 95% CI: 0.29–1.846), Google search history data (bachelor’s, OR = 0.64, 95% CI: 0.283–1.448; master’s, OR = 0.634, 95% CI: 0.278–1.449), online purchase history data (high school, OR = 6.775, 95% CI: 0.746–61.563; bachelor’s, OR = 0.72, 95% CI: 0.317–1.638; master’s, OR = 0.607, 95% CI: 0.263–1.402), credit card statement data (high school, OR = 3.52, 95% CI: 0.68–18.221; master’s, OR = 0.563, 95% CI: 0.202–1.573), and geolocation data (master’s, OR = 0.506, 95% CI: 0.215–1.194) with researchers. Notably, participants with either a bachelor’s or master’s were less likely than those with a doctorate or other terminal degree to share text message and phone data, email history data, Google search history data, online purchase history data, credit card statement data, and geolocation data. Respectively, participants with only high school education were more likely than those with a doctorate or other terminal degree to share those data sources.

### 3.7. Privacy of Personal Information Was Violated and Willingness to Donate Data

Adjusted logistical regression analyses suggest a modest relationship between participants experiencing a violation of private personal information (versus those without this experience) and willingness to share all sources of real-world data with researchers. Though none of these relationships were significant, a relatively lower willingness to share Yelp reviews and ratings data was observed (OR = 0.748, 95% CI: 0.366–1.527; see Table S10).

Within this group, adjusted logistical regression analyses identified significant relationships between participants’ age ranges and their willingness to share prescription history data (18 to 30 years, OR = 1.502, 95% CI: 0.732–3.084; 41 to 50 years, OR = 0.486, 0.233–1.014), music streaming data (18 to 30 years, OR = 9.809, 95% CI: 3.639–26.441), Google search history data (51 to 60 years, OR = 1.712, 95% CI: 0.89–3.294), tax records and income history data (31 to 40 years, OR = 1.76, 95% CI: 0.82–3.78), ridesharing history data (18 to 30 years, OR = 4.363, 95% CI: 1.654–11.51), and voting history data (18 to 30, OR = 3.209, 95% CI: 1.543–6.673). Overall, participants aged 18 to 30 years were significantly more likely than those over 60 years to share music streaming data, ridesharing history data, and voting history data, yet moderately likely to share prescription history data with researchers. Participants aged 31 to 40 years were significantly more likely than those over 60 years to share tax records and income history data, yet participants aged 41 to 50 years were significantly less likely than those over 60 years to share prescription history data with researchers. Lastly, participants aged 51 to 60 years were significantly more likely than those over 60 years to share Google search history data with researchers.

Within this group, adjusted logistical regression analyses also suggest a significant relationship between participants’ education levels and their willingness to share Twitter data (bachelor’s, OR = 0.732, 95% CI: 0.159–3.371), Snapchat data (some college/associates/trade school, OR = 0.301, 95% CI: 0.053–1.729), credit card statement data (high school, OR = 5.163, 95% CI: 0.929–28.682; master’s, OR = 0.801, 95% CI: 0.255–2.517), and geolocation data (master’s, OR = 0.5, 95% CI: 0.207–1.209) with researchers. Thus, participants with a high school education are more likely than those with a doctorate or other terminal degree to share credit card statement data. Participants with a bachelor’s degree were less likely than those with a doctorate or other terminal degree to share Twitter data, and those with some college/associates/trade school education were significantly less likely than those with a doctorate or other terminal degree to share to share Snapchat data. Lastly, participants with a master’s are less likely than those with a doctorate or other terminal degree to share credit card statement data and geolocation data with researchers.

### 3.8. Account Accessed by Someone without Permission and Willingness to Donate Data

Adjusted logistical regression analyses suggest that participants whose accounts being accessed by someone without permission were, overall, modestly or more willing to share all sources of real-world data with researchers (ranging from Yelp ratings and reviews data, OR = 0.945, CI: 0.476–1.879 to tax records and income history data, OR = 2.132, CI: 1.15–3.951; see Table S11) than those without this experience. Specifically, participants with this experience were significantly more likely than those without to share text message and phone data (OR = 1.577, CI: 0.944–2.634), tax records and income history data, credit card statement data (OR = 1.931, 95% CI: 1.033–3.608), and geolocation data (OR = 1.771, 95% CI: 1.086–2.886) with researchers.

Within this group, logistical regression analyses identified significant relationships between participants’ age ranges and their willingness to share Facebook data (41 to 50 years, OR = 0.633, 95% CI: 0.276–1.452; 51 to 60 years, OR = 1.924, 95% CI: 0.852–4.347), Twitter data (31 to 40 years, OR = 1.938, 95% CI: 0.612–6.134), prescription history data (41 to 50 years, OR = 0.436, 95% CI: 0.207–0.916), music streaming data (18 to 30 years, OR = 0. 7.393, 95% CI: 2.993–18.259), Google search history data (41 to 50 years, OR = 0.595, 95% CI: 0.282–1.258; 51 to 60 years, OR = 1.607, 95% CI: 0.841–3.07), online purchase history data (41 to 50 years, OR = 0.749, 95% CI: 0.357–1.571; 51 to 60 years, OR = 1.833, 95% CI: 0.955–3.519), tax records and income history data (31 to 40 years, OR = 1.521, 95% CI: 0.721–3.208), voting history data (18 to 30 years, OR = 2.756, 95% CI: 1.374–5.53). Overall, compared to participants over 60 years of age, those aged 18 to 30 years were significantly more likely to share music streaming data and voting history data with researchers. Those aged 31 to 40 years were significantly more likely than those over age 60 to share Twitter data and tax records and income history data with researchers, yet those aged 41 to 50 years were significantly less likely than those over age 60 to share prescription history data with researchers. Participants aged 51 to 60 years were significantly more likely than those over age 60 to share Facebook data, Google search history data, and online purchase history data with researchers. Yet, those aged 41 to 50 years were significantly less likely than those over age 60 to share Facebook data, Google search history data, and online purchase history data with researchers.

Within this group, logistical regression analyses identified significant relationships between participants’ education levels and their willingness to share Snapchat data (some college/associates/trade school, OR = 0.94, 95% CI: 0.19–4.644), email history data (master’s, OR = 0.713, 95% CI: 0.271–1.873), text message and phone data (master’s, OR = 0.612, 95% CI: 0.237–1.581), Google search history data (masters, OR = 0.719, 95% CI: 0.304–1.697), online purchase history data (master’s, OR = 0.679, 95% CI: 0.286–1.615), credit card statement data (high school, OR = 4.346, 95% CI: 0.717–26.353; master’s, OR = 0.648, 95% CI: 0.221–1.903), and geolocation data (master’s, OR = 0.48, 95% CI: 0.197–1.17). Overall, compared to those with a doctorate or other terminal degree, participants with a high school education are significantly more likely to share credit card statement data with researchers. Yet, those with a master’s degree are significantly less likely than those with a doctorate or other terminal degree to share with researchers email history data, text message and phone data, Google search history data, online purchase history data, credit card statement data, and geolocation data.

## 4. Discussion

This study is the first to explore how privacy-related experiences may shape ResearchMatch participants’ willingness to share their real-world data with researchers. This is a timely exploration of adults’ preferences to share real-world data for research, as governmental agencies within the United States, Canada, and Europe contemplate the regulatory acceptability of real-world data for innovative drugs and medical devices with precision medicine applications [33,34]. In addition, as the present study was conducted in April 2021, during the height of the COVID-19 pandemic, when privacy laws and regulations became relaxed to accommodate digital public health surveillance and remote health care, moving forward it will be important to determine if ResearchMatch participants’ privacy-related experiences and willingness to share real-world data may differ in the post-COVID era [24,35]. Overall, our increasingly complex and often opaque data privacy landscape highlights the importance of understanding what drives research participants’ choices to share their real-world data with health researchers in spite of their privacy-related experiences.

Our study found that associations between each negative privacy-related experience and participants’ willingness to share different data types were inconsistent or inconclusive, possibly limited by small sample size. Surprisingly, we did not observe that participants with negative privacy-related experience were generally unwilling to share most data sources, which is encouraging for research. Overall, it appears that participants with negative privacy-related experience were generally more likely to share non-health related data, though with exceptions. For instance, our analyses showed that participants whose reputations were negatively affected as a result of information posted online were more likely to share electronic medical record data and genetic data versus those without this experience. These observations are perhaps the most intriguing and thus useful to specifically guide precision medicine research, given the health-relatedness of these data sources. The few statistically significant findings in our study were that people with accounts accessed without permission were more likely to share credit card statement data, tax record data, and geolocation data, adjusting for age and education, than people who did not have such experience.

Further, and surprisingly, across the privacy-related experiences surveyed, participants’ age ranges and education levels corresponded with, or somehow shaped, their willingness to share certain data sources with researchers. Notably, across all privacy-related experiences, participants aged 18 to 30 were significantly more likely than those over 60 years to share music streaming data, ridesharing history data, and voting history data.

Interestingly, our analyses showed that participants with a master’s degree were unwilling to share most sources of data, compared to those with a doctorate or terminal degree, in the event they experienced any of the five privacy-related experiences. Reasons for this observation might center on the idea that, and past work describing how, those with a doctorate or other terminal degree might be naturally inclined to indicate a general willingness to share data in general for research [36]. This is especially given the research-driven nature of many doctoral professions as well as researchers’ perceived advantages to sharing data outweighing perceived disadvantages [37]. On the contrary, however, our analyses showed that, among participants who had an unpleasant experience as a result of giving out information online, those with some college/associates/trade school compared to those with a doctoral or other terminal degree were significantly more willing to share genetic data. Furthermore, across all privacy-related experiences, those with a high school education were significantly more likely than those with a doctorate or other terminal degree to share credit card statement data. Future research should explore potential underlying reasons for these observations within the ResearchMatch population, as well as determine whether similar effects can be observed among adults outside of ResearchMatch. We anticipate that these observations may open new areas of inquiry to explore real-world factors that motivate the likelihood of research participants sharing specific data with researchers.

Our study is accompanied by limitations. First, some of our logistical regression results were uninterpretable due to relatively low numbers of responses and/or missing data not at random (i.e., willingness to share Snapchat and Yelp ratings and reviews data, perhaps due to the low utilization of these data sources in our survey sample). Future work should examine and confirm our observations purposively among ResearchMatch participants by engaging a larger survey sample size of adults and controlling for potential confounders. Additionally, older adults over age 51 years comprised the majority (49% total) of our survey respondents, as did those with a master’s or bachelor’s degree (63% total). Future work should also explore and confirm our findings among ResearchMatch participants 50 years of age and under and/or with a high school diploma, some college/associates/trade school, and doctorate or other terminal degree levels of education. Such work would further assist researchers and ResearchMatch participants across this range of demographics as they prepare to engage in personalized medicine research involving one or more of the real-world data sources assessed in our present study.

## 5. Conclusions

Our study is the first to explore how privacy-related experiences, age, and education levels may shape ResearchMatch participants’ willingness to share 19 different real-world data sources (social media data, health data, direct communication data, online browsing or streaming data, financial data, location data, and voting history data) with researchers. Altogether, our findings open new opportunities to engage ResearchMatch participants in studies that further examine their real-world data sharing preferences with researchers. In addition, given these observations, opportunities to balance ResearchMatch participants’ health research engagement preferences, based on their lived privacy-related experiences, are critical to the success of precision or personalized medicine research within and outside of regulatory settings.

## Data Availability

The data presented in this study are available on request from the corresponding author. The data are not publicly available to protect the privacy of research participants.

## Supplementary Materials

The following supporting information can be downloaded at: https://www.mdpi.com/article/10.3390/jpm12111922/s1, Table S1: Summary of survey respondents’ demographics (excluding non-responses); Table S2: Participants’ willingness to donate data sources who have been victims of fraud and/or identity theft online; Table S3: Participants’ willingness to donate data sources who have had an unpleasant experience as a result of information given out online; Table S4: Participants’ willingness to donate data sources whose reputation was negatively affected as a result of information posted online; Table S5: Participants’ willingness to donate data sources whose privacy of personal information was violated; Table S6: Participants’ willingness to donate data sources whose account accessed by someone without permission; Table S7: Associations between willingness to share real-world data from various sources and experienced being victim of fraud and/or identity theft, adjusted for age range and education level; Table S8: Associations between willingness to share real-world data from various sources and had an unpleasant experience as a result of information posted online, adjusted for age range and education level; Table S9: Associations between willingness to share real-world data from various sources and experienced reputation being negatively affected as a result of information posted online, adjusted for age range and education level; Table S10: Associations between willingness to share real-world data from various sources and experienced privacy of personal information being violated, adjusted for age range and education level; Table S11: Associations between willingness to share real-world data from various sources and experienced account being accessed without permission, adjusted for age range and education level.
