# Supplementary material for: A Survey of Research Participants’ Privacy-Related Experiences and Willingness to Share Real-World Data with Researchers
Source: J Pers Med. 2022 Nov 17;12(11):1922. doi: 10.3390/jpm12111922 (PMC9696408; doi:10.3390/jpm12111922)
Supplement: Supplementary file 1 [file jpm-12-01922-s001.zip › Table S10_Privacy of Personal Information was Violated.pdf]

Table S10. Associations between willingness to share real-world data from various sources and experienced privacy of personal information being violated, adjusted for age range and education level.

**Real-World Data Source**	**Logistic regression results**			
**Social Media Data**				
Facebook data (n= 243)	***Adjusted Odds Ratio***	***95% confidence interval***		***P-Value***
Privacy of personal information being violated	0.875	0.509	1.506	0.6303
Age range (ref = over 60)				
18 to 30	1.152	0.494	2.686	0.6037
31 to 40	0.81	0.376	1.746	0.4499
41 to 50	0.633	0.274	1.462	0.1317
51 to 60	1.584	0.711	3.529	0.0902*
Education (ref = Doctorate or other terminal degree)				
High school	5.985	0.603	59.415	0.1204
Some College/Associates/Trade School	1.233	0.434	3.502	0.4846
Bachelors	1.301	0.469	3.612	0.5795
Masters	0.922	0.332	2.563	0.0987*
Twitter data (n= 122)	***Adjusted Odds Ratio***	***95% confidence interval***		***P-Value***
Privacy of personal information being violated	1.132	0.487	2.631	0.2523
Age range (ref = over 60)				
18 to 30	4.476	1.243	16.114	0.7739
31 to 40	2.975	0.936	9.455	0.1588
41 to 50	1.405	0.396	4.982	0.5904
51 to 60	4.575	1.256	16.663	0.1928
Education (ref = Doctorate or other terminal degree)				
High school	0.949	0.064	14.058	0.1527
Some College/Associates/Trade School	0.28	0.057	1.372	0.6638
Bachelors	0.732	0.159	3.371	0.0723*
Masters	0.478	0.1	2.287	0.6851
Instagram data (n= 173)	***Adjusted Odds Ratio***	***95% confidence interval***		***P-Value***
Privacy of personal information being violated	0.825	0.426	1.597	0.5677
Age range (ref = over 60)				
18 to 30	1.755	0.651	4.73	0.7635

31 to 40	1.515	0.577	3.98	0.855
41 to 50	1.969	0.688	5.64	0.5377
51 to 60	1.993	0.656	6.055	0.5549
Education (ref = Doctorate or other terminal degree)				
High school	2.863	0.233	35.101	0.5454
Some College/Associates/Trade School	1.403	0.402	4.897	0.7077
Bachelors	2.048	0.608	6.904	0.509
Masters	1.345	0.404	4.48	0.5999
Snapchat data (n= 103)	***Adjusted Odds Ratio***	***95% confidence interval***		***P-Value***
Privacy of personal information being violated	1.186	0.48	2.93	0.7122
Age range (ref = over 60)				
18 to 30	-	-	-	-
31 to 40	-	-	-	-
41 to 50	-	-	-	-
51 to 60	-	-	-	-
Education (ref = Doctorate or other terminal degree)				
High school	1.158	0.048	28.047	0.754
Some College/Associates/Trade School	0.301	0.053	1.729	0.0764*
Bachelors	0.959	0.199	4.636	0.6866
Masters	0.977	0.186	5.127	0.6869
Yelp reviews and ratings data (n= 168)	***Adjusted Odds Ratio***	***95% confidence interval***		***P-Value***
Privacy of personal information being violated	0.748	0.366	1.527	0.4251
Age range (ref = over 60)				
18 to 30	2.484	0.828	7.454	0.6084
31 to 40	1.747	0.703	4.342	0.6593
41 to 50	2.062	0.792	5.37	0.9497
51 to 60	3.732	1.324	10.523	0.1109
Education (ref = Doctorate or other terminal degree)				
High school	-	-	-	-
Some College/Associates/Trade School	0.996	0.29	3.417	0.977
Bachelors	1.107	0.326	3.758	0.9779
Masters	1.191	0.347	4.087	0.9786
**Health Data**				

Fitness tracker data (n= 212)	*Adjusted Odds Ratio*	*95% confidence interval*		*P-Value*
Privacy of personal information being violated	0.84	0.43	1.644	0.6114
Age range (ref = over 60)				
18 to 30	3.044	1.046	8.853	0.2099
31 to 40	1.364	0.596	3.122	0.3211
41 to 50	1.579	0.645	3.863	0.6497
51 to 60	3.176	1.117	9.027	0.1655
Education (ref = Doctorate or other terminal degree)				
High school	0.613	0.081	4.646	0.5574
Some College/Associates/Trade School	0.999	0.341	2.924	0.91
Bachelors	1.252	0.431	3.635	0.4331
Masters	1.068	0.383	2.978	0.7378
Prescription history data (n= 317)	***Adjusted Odds Ratio***	***95% confidence interval***		***P-Value***
Privacy of personal information being violated	0.797	0.499	1.273	0.3416
Age range (ref = over 60)				
18 to 30	1.502	0.732	3.084	0.0447**
31 to 40	0.832	0.436	1.585	0.7919
41 to 50	0.486	0.233	1.014	0.0274**
51 to 60	0.891	0.465	1.707	0.9754
Education (ref = Doctorate or other terminal degree)				
High school	1.258	0.283	5.589	0.9486
Some College/Associates/Trade School	1.459	0.593	3.588	0.4613
Bachelors	1.486	0.621	3.556	0.3838
Masters	0.973	0.402	2.358	0.3522
Electronic medical record data (n= 317)	***Adjusted Odds Ratio***	***95% confidence interval***		***P-Value***
Privacy of personal information being violated	0.856	0.536	1.368	0.5158
Age range (ref = over 60)				
18 to 30	0.441	0.217	0.897	0.2467
31 to 40	0.656	0.341	1.26	0.6792
41 to 50	0.464	0.225	0.96	0.3503
51 to 60	0.556	0.288	1.076	0.7746
Education (ref = Doctorate or other terminal degree)				
High school	0.744	0.143	3.857	0.7923

Some College/Associates/Trade School	0.78	0.317	1.917	0.6653
Bachelors	1.1	0.461	2.621	0.3326
Masters	0.791	0.327	1.91	0.6949
**Genetic data (n= 227)**	**Adjusted Odds Ratio**	**95% confidence interval**		**P-Value**
Privacy of personal information being violated	0.856	0.493	1.487	0.5809
Age range (ref = over 60)				
18 to 30	0.622	0.267	1.446	0.6749
31 to 40	0.601	0.269	1.344	0.5731
41 to 50	0.51	0.23	1.132	0.2572
51 to 60	0.935	0.427	2.048	0.3259
Education (ref = Doctorate or other terminal degree)				
High school	0.407	0.063	2.616	0.3384
Some College/Associates/Trade School	0.985	0.364	2.663	0.4629
Bachelors	0.918	0.353	2.384	0.5953
Masters	0.841	0.315	2.242	0.8345
**Direct Communication Data**				
Text message and phone data (n= 317)	***Adjusted Odds Ratio***	***95% confidence interval***		***P-Value***
Privacy of personal information being violated	0.945	0.572	1.56	0.8244
Age range (ref = over 60)				
18 to 30	0.913	0.423	1.974	0.663
31 to 40	1.429	0.722	2.829	0.1732
41 to 50	0.873	0.392	1.948	0.5698
51 to 60	1.024	0.504	2.08	0.9777
Education (ref = Doctorate or other terminal degree)				
High school	1.253	0.246	6.385	0.8363
Some College/Associates/Trade School	1.211	0.461	3.183	0.7439
Bachelors	1.272	0.498	3.25	0.5819
Masters	0.876	0.334	2.294	0.3688
**Email history data (n= 319)**	**Adjusted Odds Ratio**	**95% confidence interval**		**P-Value**
Privacy of personal information being violated	0.832	0.505	1.37	0.4698
Age range (ref = over 60)				
18 to 30	0.747	0.348	1.604	0.3551
31 to 40	1.093	0.546	2.188	0.6199

41 to 50	0.82	0.373	1.803	0.5688
51 to 60	1.263	0.638	2.5	0.2728
Education (ref = Doctorate or other terminal degree)				
High school	2.173	0.465	10.155	0.5374
Some College/Associates/Trade School	2.318	0.877	6.126	0.115
Bachelors	1.85	0.713	4.801	0.4731
Masters	0.987	0.365	2.668	0.0858*
**Online Browsing or Streaming Data**				
Music streaming data (n= 245)	***Adjusted Odds Ratio***	***95% confidence interval***		***P-Value***
Privacy of personal information being violated	0.868	0.485	1.553	0.633
Age range (ref = over 60)				
18 to 30	9.809	3.639	26.441	0.0017**
31 to 40	2.977	1.349	6.57	0.6942
41 to 50	2.567	1.098	6.001	0.3916
51 to 60	5.223	2.099	12.993	0.144
Education (ref = Doctorate or other terminal degree)				
High school	0.883	0.123	6.322	0.8104
Some College/Associates/Trade School	1.544	0.539	4.425	0.2448
Bachelors	0.789	0.293	2.124	0.3255
Masters	1.209	0.438	3.336	0.6544
**Google search history data (n= 323)**	**Adjusted Odds Ratio**	**95% confidence interval**		**P-Value**
Privacy of personal information being violated	0.977	0.614	1.555	0.9227
Age range (ref = over 60)				
18 to 30	0.968	0.478	1.962	0.6484
31 to 40	1.032	0.538	1.977	0.8174
41 to 50	0.892	0.434	1.834	0.4489
51 to 60	1.712	0.89	3.294	0.0514**
Education (ref = Doctorate or other terminal degree)				
High school	3.364	0.708	15.985	0.1024
Some College/Associates/Trade School	1.383	0.578	3.313	0.8642
Bachelors	0.924	0.396	2.156	0.124
Masters	0.951	0.402	2.247	0.172

**Financial Data**				
Online purchase history data (n= 319)	***Adjusted Odds Ratio***	***95% confidence interval***		***P-Value***
Privacy of personal information being violated	0.803	0.505	1.277	0.3536
Age range (ref = over 60)				
18 to 30	1.445	0.717	2.912	0.8426
31 to 40	1.648	0.86	3.16	0.4271
41 to 50	1.051	0.508	2.172	0.3065
51 to 60	1.961	1.01	3.81	0.1298
Education (ref = Doctorate or other terminal degree)				
High school	3.833	0.663	22.174	0.1022*
Some College/Associates/Trade School	1.085	0.451	2.611	0.475
Bachelors	1.019	0.436	2.382	0.3079
Masters	0.911	0.383	2.166	0.1585
Tax records and income history data (n= 314)	***Adjusted Odds Ratio***	***95% confidence interval***		***P-Value***
Privacy of personal information being violated	1.23	0.678	2.23	0.4955
Age range (ref = over 60)				
18 to 30	1.175	0.487	2.83	0.6917
31 to 40	1.76	0.82	3.78	0.045**
41 to 50	0.707	0.256	1.951	0.3131
51 to 60	0.815	0.34	1.953	0.4451
Education (ref = Doctorate or other terminal degree)				
High school	3.079	0.481	19.708	0.1179
Some College/Associates/Trade School	0.812	0.288	2.293	0.3943
Bachelors	0.692	0.252	1.9	0.1413
Masters	0.762	0.274	2.117	0.2785
Credit card statement data (n=306)	***Adjusted Odds Ratio***	***95% confidence interval***		***P-Value***
Privacy of personal information being violated	0.942	0.515	1.722	0.8457
Age range (ref = over 60)				
Indent 18 to 30	0.637	0.259	1.569	0.673
Indent 31 to 40	0.877	0.387	1.991	0.5578
Indent 41 to 50	0.68	0.261	1.77	0.8307
Indent 51 to 60	0.563	0.234	1.353	0.4162
Education				

High school	5.163	0.929	28.682	0.0316**
Some College/Associates/Trade School	1.189	0.378	3.739	0.5364
Bachelors	1.25	0.414	3.777	0.6169
Masters	0.801	0.255	2.517	0.0592*
**Location Data**				
Ridesharing history data (n= 178)	***Adjusted Odds Ratio***	***95% confidence interval***		***P-Value***
Privacy of personal information being violated	0.874	0.462	1.651	0.678
Age range (ref = over 60)				
18 to 30	4.363	1.654	11.51	0.0484**
31 to 40	2.246	0.884	5.706	0.8726
41 to 50	2.419	0.859	6.814	0.9397
51 to 60	3.061	1.103	8.496	0.4427
Education (ref = Doctorate or other terminal degree)				
High school	2.158	0.268	17.355	0.535
Some College/Associates/Trade School	1.631	0.517	5.142	0.5734
Bachelors	1.091	0.375	3.174	0.5378
Masters	1.081	0.361	3.239	0.5324
Geolocation data (n= 312)	***Adjusted Odds Ratio***	***95% confidence interval***		***P-Value***
Privacy of personal information being violated	0.899	0.557	1.452	0.6646
Age range (ref = over 60)				
18 to 30	0.88	0.426	1.815	0.7817
31 to 40	1.024	0.525	1.998	0.7341
41 to 50	0.657	0.307	1.407	0.1877
51 to 60	1.275	0.646	2.518	0.2133
Education (ref = Doctorate or other terminal degree)				
High school	1.253	0.252	6.214	0.5478
Some College/Associates/Trade School	1.096	0.455	2.636	0.3871
Bachelors	0.766	0.326	1.801	0.5644
Masters	0.5	0.207	1.209	0.0285**
**Voting History Data (n= 309)**	***Adjusted Odds Ratio***	***95% confidence interval***		***P-Value***
Privacy of personal information being violated	1.075	0.669	1.727	0.7661
Age range (ref = over 60)				
18 to 30	3.209	1.543	6.673	0.0046**

31 to 40	1.447	0.751	2.788	0.8645
41 to 50	1.419	0.674	2.99	0.8293
51 to 60	1.173	0.601	2.289	0.2965
Education (ref = Doctorate or other terminal degree)				
High school	1.403	0.276	7.133	0.9974
Some College/Associates/Trade School	1.854	0.738	4.659	0.2775
Bachelors	1.478	0.603	3.622	0.8243
Masters	1.4	0.569	3.445	0.9996

**Significant value ($p \leq 0.05$)

*Modestly significant value ($p \leq 0.10$)
