# Supplementary material for: A Survey of Research Participants’ Privacy-Related Experiences and Willingness to Share Real-World Data with Researchers
Source: J Pers Med. 2022 Nov 17;12(11):1922. doi: 10.3390/jpm12111922 (PMC9696408; doi:10.3390/jpm12111922)
Supplement: Supplementary file 1 [file jpm-12-01922-s001.zip › Table S11_Account Accessed wo permission.pdf]

Table S11. Associations between willingness to share real-world data from various sources and experienced account being accessed without permission, adjusted for age range and education level.

Real-World Data Source	Logistic regression results			
**Social Media Data**				
Facebook data (n= 253)	***Adjusted Odds Ratio***	***95% confidence interval***		***P-Value***
Account accessed without permission	1.051	0.607	1.819	0.8601
Age range (ref = over 60)				
18 to 30	1.412	0.636	3.134	0.3235
31 to 40	0.816	0.38	1.751	0.303
41 to 50	0.633	0.276	1.452	0.0764*
51 to 60	1.924	0.852	4.347	0.0428**
Education (ref = Doctorate or other terminal degree)				
High school	4.58	0.432	48.507	0.2256
Some College/Associates/Trade School	1.449	0.502	4.183	0.8476
Bachelors	1.475	0.526	4.14	0.884
Masters	0.898	0.318	2.53	0.0849*
Twitter data (n= 123)	***Adjusted Odds Ratio***	***95% confidence interval***		***P-Value***
Account accessed without permission	1.99	0.839	4.72	0.6351
Age range (ref = over 60)				
18 to 30	6.665	1.75	25.381	0.1182
31 to 40	1.938	0.612	6.134	0.0256**
41 to 50	1.13	0.315	4.062	0.5996
51 to 60	5.01	1.327	18.918	0.0981*
Education (ref = Doctorate or other terminal degree)				
High school	0.738	0.044	12.464	0.1051
Some College/Associates/Trade School	0.252	0.051	1.25	0.7781
Bachelors	0.657	0.141	3.053	0.0878*
Masters	0.417	0.086	2.009	0.6772
Instagram data (n= 183)	***Adjusted Odds Ratio***	***95% confidence interval***		***P-Value***
Account accessed without permission	1.023	0.528	1.983	0.9454
Age range (ref = over 60)				
18 to 30	2.352	0.915	6.044	0.3159

31 to 40	1.583	0.618	4.057	0.7211
41 to 50	1.934	0.677	5.525	0.7748
51 to 60	2.298	0.77	6.857	0.4598
Education (ref = Doctorate or other terminal degree)				
High school	2.72	0.22	33.615	0.4982
Some College/Associates/Trade School	1.409	0.419	4.743	0.9697
Bachelors	1.658	0.517	5.321	0.6671
Masters	0.939	0.287	3.068	0.2334
Snapchat data (n= 107)	***Adjusted Odds Ratio***	***95% confidence interval***		***P-Value***
Account accessed without permission	-	-	-	-
Age range (ref = over 60)				
18 to 30	-	-	-	-
31 to 40	-	-	-	-
41 to 50	-	-	-	-
51 to 60	1.784	0.07	45.577	0.976
Education (ref = Doctorate or other terminal degree)				
High school	0.298	0.051	1.73	0.5663
Some College/Associates/Trade School	0.94	0.19	4.644	0.0471**
Bachelors	1.145	0.209	6.267	0.9131
Masters	-	-	-	-
Yelp reviews and ratings data (n= 173)	***Adjusted Odds Ratio***	***95% confidence interval***		***P-Value***
Account accessed without permission	0.945	0.476	1.879	0.8727
Age range (ref = over 60)				
18 to 30	1.948	0.69	5.494	0.912
31 to 40	1.629	0.642	4.132	0.69
41 to 50	2.228	0.86	5.774	0.6196
51 to 60	3.203	1.147	8.942	0.1636
Education (ref = Doctorate or other terminal degree)				
High school	-	-	-	-
Some College/Associates/Trade School	0.698	0.2	2.428	0.9632
Bachelors	1.011	0.292	3.496	0.9686
Masters	0.911	0.263	3.156	0.9671
**Health Data**				

Fitness tracker data (n= 214)	***Adjusted Odds Ratio***	***95% confidence interval***		***P-Value***
Account accessed without permission	1.607	0.835	3.091	0.1554
Age range (ref = over 60)				
18 to 30	3.549	1.165	10.805	0.1025*
31 to 40	1.275	0.549	2.964	0.2866
41 to 50	1.357	0.536	3.434	0.4423
51 to 60	2.871	1.016	8.112	0.2228
Education (ref = Doctorate or other terminal degree)				
High school	0.8	0.102	6.273	0.7199
Some College/Associates/Trade School	1.034	0.355	3.012	0.952
Bachelors	1.5	0.511	4.399	0.3096
Masters	1.057	0.377	2.965	0.996
Prescription history data (n= 334)	***Adjusted Odds Ratio***	***95% confidence interval***		***P-Value***
Account accessed without permission	1.052	0.665	1.665	0.8286
Age range (ref = over 60)				
18 to 30	1.234	0.627	2.43	0.1*
31 to 40	0.823	0.436	1.553	0.9914
41 to 50	0.436	0.207	0.916	0.0219**
51 to 60	0.842	0.444	1.597	0.9119
Education (ref = Doctorate or other terminal degree)				
High school	0.924	0.197	4.34	0.843
Some College/Associates/Trade School	1.238	0.512	2.993	0.4598
Bachelors	1.368	0.58	3.226	0.2216
Masters	0.749	0.314	1.789	0.1787
Electronic medical record data (n= 336)	***Adjusted Odds Ratio***	***95% confidence interval***		***P-Value***
Account accessed without permission	1.125	0.71	1.783	0.6152
Age range (ref = over 60)				
18 to 30	0.514	0.263	1.004	0.5254
31 to 40	0.645	0.341	1.22	0.748
41 to 50	0.436	0.21	0.905	0.2373
51 to 60	0.535	0.28	1.024	0.6307
Education (ref = Doctorate or other terminal degree)				
High school	0.705	0.137	3.625	0.8389

Some College/Associates/Trade School	0.733	0.306	1.752	0.7394
Bachelors	1.004	0.432	2.332	0.3252
Masters	0.619	0.262	1.466	0.3064
**Genetic data (n= 239)**	**Adjusted Odds Ratio**	**95% confidence interval**		**P-Value**
Account accessed without permission	1.578	0.916	2.716	0.0999*
Age range (ref = over 60)				
18 to 30	0.596	0.267	1.328	0.6417
31 to 40	0.635	0.284	1.418	0.8028
41 to 50	0.494	0.218	1.115	0.2781
51 to 60	0.795	0.367	1.725	0.586
Education (ref = Doctorate or other terminal degree)				
High school	0.469	0.072	3.062	0.4191
Some College/Associates/Trade School	1.123	0.414	3.048	0.2945
Bachelors	1.156	0.443	3.016	0.2212
Masters	0.622	0.231	1.678	0.3409
**Direct Communication Data**				
Text message and phone data (n= 335)	***Adjusted Odds Ratio***	***95% confidence interval***		***P-Value***
Account accessed without permission	1.577	0.944	2.634	0.0819*
Age range (ref = over 60)				
18 to 30	1.019	0.492	2.11	0.7988
31 to 40	1.181	0.599	2.33	0.3721
41 to 50	0.628	0.27	1.461	0.182
51 to 60	1.04	0.519	2.084	0.7244
Education (ref = Doctorate or other terminal degree)				
High school	2.263	0.447	11.457	0.1971
Some College/Associates/Trade School	1.091	0.429	2.776	0.918
Bachelors	0.897	0.36	2.236	0.4862
Masters	0.612	0.237	1.581	0.0379**
**Email history data (n= 337)**	**Adjusted Odds Ratio**	**95% confidence interval**		**P-Value**
Account accessed without permission	1.261	0.767	2.074	0.3601
Age range (ref = over 60)				
18 to 30	0.79	0.386	1.617	0.7687
31 to 40	0.824	0.414	1.639	0.8901

41 to 50	0.573	0.253	1.297	0.1915
51 to 60	1.21	0.62	2.359	0.1461
Education				
High school	3.625	0.747	17.594	0.1105
Some College/Associates/Trade School	1.919	0.756	4.874	0.2933
Bachelors	1.414	0.566	3.533	0.8564
Masters	0.713	0.271	1.873	0.0064**
**Online Browsing or Streaming Data**				
Music streaming data (n= 261)	***Adjusted Odds Ratio***	***95% confidence interval***		***P-Value***
Account accessed without permission	1.492	0.846	2.628	0.1666
Age range (ref = over 60)				
18 to 30	7.393	2.993	18.259	0.0032**
31 to 40	2.744	1.283	5.868	0.8879
41 to 50	2.138	0.931	4.91	0.3355
51 to 60	4.31	1.795	10.35	0.1807
Education (ref = Doctorate or other terminal degree)				
High school	1.163	0.165	8.187	0.9728
Some College/Associates/Trade School	1.386	0.494	3.888	0.5265
Bachelors	1.002	0.377	2.666	0.665
Masters	1.166	0.426	3.192	0.9288
**Google search history data (n= 340)**	**Adjusted Odds Ratio**	**95% confidence interval**		**P-Value**
Account accessed without permission	1.468	0.92	2.342	0.1072
Age range (ref = over 60)				
18 to 30	0.909	0.462	1.788	0.8183
31 to 40	0.945	0.497	1.798	0.942
41 to 50	0.595	0.282	1.258	0.0831*
51 to 60	1.607	0.841	3.07	0.0277**
Education (ref = Doctorate or other terminal degree)				
High school	2.834	0.565	14.214	0.1325
Some College/Associates/Trade School	1.337	0.561	3.185	0.5736
Bachelors	0.775	0.333	1.8	0.0855*
Masters	0.719	0.304	1.697	0.0504**

Financial Data				
Online purchase history data (n= 337)	***Adjusted Odds Ratio***	***95% confidence interval***		***P-Value***
Account accessed without permission	1.279	0.807	2.027	0.2951
Age range (ref = over 60)				
18 to 30	1.488	0.765	2.894	0.3919
31 to 40	1.282	0.68	2.418	0.8033
41 to 50	0.749	0.357	1.571	0.077*
51 to 60	1.833	0.955	3.519	0.0774*
Education (ref = Doctorate or other terminal degree)				
High school	2.953	0.493	17.685	0.1464
Some College/Associates/Trade School	1.034	0.432	2.478	0.7786
Bachelors	0.825	0.355	1.921	0.2219
Masters	0.679	0.286	1.615	0.0551*
Tax records and income history data (n= 331)	***Adjusted Odds Ratio***	***95% confidence interval***		***P-Value***
Account accessed without permission	2.132	1.15	3.951	0.0162**
Age range (ref = over 60)				
18 to 30	1.006	0.437	2.316	0.8981
31 to 40	1.521	0.721	3.208	0.0815*
41 to 50	0.807	0.32	2.038	0.595
51 to 60	0.688	0.289	1.638	0.2764
Education (ref = Doctorate or other terminal degree)				
High school	2.242	0.318	15.812	0.3081
Some College/Associates/Trade School	0.939	0.338	2.609	0.6751
Bachelors	0.79	0.292	2.139	0.2977
Masters	0.834	0.303	2.298	0.4127
Credit card statement data (n=324)	***Adjusted Odds Ratio***	***95% confidence interval***		***P-Value***
Account accessed without permission	1.931	1.033	3.608	0.0393**
Age range (ref = over 60)				
Indent 18 to 30	0.585	0.249	1.373	0.6735
Indent 31 to 40	0.676	0.303	1.511	0.9709
Indent 41 to 50	0.76	0.314	1.842	0.7036
Indent 51 to 60	0.446	0.186	1.072	0.2185

Education				
High school	4.346	0.717	26.353	0.0524**
Some College/Associates/Trade School	1.019	0.351	2.958	0.5204
Bachelors	0.997	0.353	2.814	0.4452
Masters	0.648	0.221	1.903	0.035**
**Location Data**				
Ridesharing history data (n= 189)	***Adjusted Odds Ratio***	***95% confidence interval***		***P-Value***
Account accessed without permission	1.081	0.578	2.021	0.8082
Age range (ref = over 60)				
18 to 30	3.243	1.324	7.944	0.1417
31 to 40	2.073	0.825	5.207	0.9195
41 to 50	2.113	0.776	5.759	0.9754
51 to 60	3.129	1.131	8.655	0.2689
Education (ref = Doctorate or other terminal degree)				
High school	3.009	0.367	24.688	0.3955
Some College/Associates/Trade School	1.593	0.496	5.112	0.9462
Bachelors	1.563	0.528	4.621	0.9879
Masters	1.215	0.394	3.745	0.4479
Geolocation data (n= 325)	***Adjusted Odds Ratio***	***95% confidence interval***		***P-Value***
Account accessed without permission	1.771	1.086	2.886	0.0219**
Age range (ref = over 60)				
18 to 30	1.205	0.603	2.41	0.5783
31 to 40	1.134	0.58	2.221	0.7468
41 to 50	0.671	0.309	1.457	0.11
51 to 60	1.4	0.706	2.775	0.2376
Education (ref = Doctorate or other terminal degree)				
High school	3.405	0.536	21.644	0.1039*
Some College/Associates/Trade School	1.146	0.476	2.76	0.8617
Bachelors	0.835	0.354	1.966	0.2902
Masters	0.48	0.197	1.17	0.0027**
**Voting History Data (n= 327)**	***Adjusted Odds Ratio***	***95% confidence interval***		***P-Value***
Account accessed without permission	1.434	0.9	2.286	0.1292

Age range (ref = over 60)				
18 to 30	2.756	c	5.53	0.0152**
31 to 40	1.457	0.765	2.773	0.9208
41 to 50	1.474	0.705	3.08	0.9669
51 to 60	1.243	0.644	2.399	0.442
Education (ref = Doctorate or other terminal degree)				
High school	1.095	0.198	6.047	0.8178
Some College/Associates/Trade School	1.616	0.655	3.985	0.3484
Bachelors	1.674	0.697	4.017	0.251
Masters	1.1	0.453	2.671	0.5757

**Significant value ($p \leq 0.05$)

*Modestly significant value ($p \leq 0.10$)
