# Supplementary material for: A Survey of Research Participants’ Privacy-Related Experiences and Willingness to Share Real-World Data with Researchers
Source: J Pers Med. 2022 Nov 17;12(11):1922. doi: 10.3390/jpm12111922 (PMC9696408; doi:10.3390/jpm12111922)
Supplement: Supplementary file 1 [file jpm-12-01922-s001.zip › Table S1_Summary of survey respondents demographics .pdf]

Table S1. Summary of survey respondents' demographics (excluding non-responses).

	**N (%) respondents**
**Age Range (years)**	
18 to 30	66 (17%)
31 to 40	78 (19%)
41 to 50	57 (14%)
51 to 60	71 (18%)
Over 60	126 (31%)
**Education Level**	
High School	12 (3%)
Some College/Associates/Trade School	98 (24%)
Bachelors	138 (34%)
Masters	117 (29%)
Doctorate or other terminal degree	35 (9%)
**Duration of using online medical websites (years)**	
Less than 1	18 (4%)
2 to 3	36 (9%)
4 to 5	53 (13%)
6 to 7	38 (9%)
More than 7	224 (56%)
Never	5 (1%)
Unsure	26 (6%)
**Annual frequency of getting ill (number of occurrences)**	
Less than 1	193 (48%)
2 to 3	149 (37%)
4 to 6	35 (9%)
7 to 10	5 (1%)
More than 10	16 (4%)
