# Supplementary material for: A Survey of Research Participants’ Privacy-Related Experiences and Willingness to Share Real-World Data with Researchers
Source: J Pers Med. 2022 Nov 17;12(11):1922. doi: 10.3390/jpm12111922 (PMC9696408; doi:10.3390/jpm12111922)
Supplement: Supplementary file 1 [file jpm-12-01922-s001.zip › Table S7_victim of fraud and_or identity theft.pdf]

Table S7. Associations between willingness to share real-world data from various sources and experienced being victim of fraud and/or identity theft, adjusted for age range and education level.

**Real-World Data Source**	**Logistic regression results**			
**Social Media Data**				
Facebook data (n= 270)	***Adjusted Odds Ratio***	***95% confidence interval***		***P-Value***
Victim of fraud and/or identity theft	0.729	0.43	1.235	0.2395
Age range (ref = over 60)				
18 to 30	1.274	0.569	2.852	0.5827
31 to 40	1.004	0.473	2.132	0.7419
41 to 50	0.756	0.345	1.657	0.1865
51 to 60	1.61	0.734	3.531	0.1586
Education (ref = Doctorate or other terminal degree)				
High school	2.033	0.323	12.817	0.3577
Some College/Associates/Trade School	1.112	0.394	3.137	0.9749
Bachelors	0.931	0.341	2.539	0.5289
Masters	0.772	0.281	2.125	0.1929
Twitter data (n= 131)	***Adjusted Odds Ratio***	***95% confidence interval***		***P-Value***
Victim of fraud and/or identity theft	0.794	0.34	1.854	0.5937
Age range (ref = over 60)				
18 to 30	3.241	0.887	11.852	0.3347
31 to 40	2.248	0.689	7.337	0.8963
41 to 50	1.393	0.421	4.611	0.2915
51 to 60	4.4	1.173	16.507	0.1172
Education (ref = Doctorate or other terminal degree)				
High school	0.839	0.054	13.069	0.5611
Some College/Associates/Trade School	0.191	0.033	1.099	0.0327**
Bachelors	0.555	0.101	3.05	0.7391
Masters	0.3	0.054	1.677	0.2517
Instagram data (n= 196)	***Adjusted Odds Ratio***	***95% confidence interval***		***P-Value***
Victim of fraud and/or identity theft	0.633	0.337	1.187	0.1539
Age range (ref = over 60)				
18 to 30	1.388	0.523	3.685	0.8878

31 to 40	1.358	0.53	3.48	0.8207
41 to 50	1.871	0.675	5.186	0.4353
51 to 60	1.804	0.61	5.333	0.5456
Education (ref = Doctorate or other terminal degree)				
High school	2.133	0.186	24.407	0.5189
Some College/Associates/Trade School	1.057	0.309	3.613	0.7597
Bachelors	1.345	0.415	4.365	0.7059
Masters	0.764	0.237	2.468	0.1954
Snapchat data (n= 110)	***Adjusted Odds Ratio***	***95% confidence interval***		***P-Value***
Victim of fraud and/or identity theft	1.383	0.57	3.354	0.4735
Age range (ref = over 60)				
18 to 30	-	-	-	-
31 to 40	-	-	-	-
41 to 50	-	-	-	-
51 to 60	-	-	-	-
Education (ref = Doctorate or other terminal degree)				
High school	0.665	0.028	15.755	0.8498
Some College/Associates/Trade School	0.2	0.034	1.186	0.065*
Bachelors	0.506	0.101	2.551	0.908
Masters	0.636	0.117	3.471	0.7228
Yelp reviews and ratings data (n= 185)	***Adjusted Odds Ratio***	***95% confidence interval***		***P-Value***
Victim of fraud and/or identity theft	0.793	0.416	1.513	0.4823
Age range (ref = over 60)				
18 to 30	1.989	0.705	5.609	0.9448
31 to 40	2.056	0.812	5.205	0.859
41 to 50	2.158	0.874	5.328	0.7469
51 to 60	3.1	1.162	8.268	0.1961
Education (ref = Doctorate or other terminal degree)				
High school	-	-	-	-
Some College/Associates/Trade School	1.07	0.322	3.551	0.9781
Bachelors	1.075	0.327	3.531	0.9782
Masters	0.999	0.309	3.228	0.9775

**Health Data**				
Fitness tracker data (n= 226)	***Adjusted Odds Ratio***	***95% confidence interval***		***P-Value***
Victim of fraud and/or identity theft	1.032	0.547	1.948	0.9221
Age range (ref = over 60)				
18 to 30	3.27	1.121	9.539	0.1842
31 to 40	1.537	0.66	3.575	0.4706
41 to 50	1.713	0.696	4.217	0.7384
51 to 60	3.022	1.075	8.494	0.2486
Education (ref = Doctorate or other terminal degree)				
High school	0.671	0.094	4.81	0.687
Some College/Associates/Trade School	0.871	0.291	2.606	0.9195
Bachelors	1.189	0.397	3.567	0.4163
Masters	0.856	0.305	2.398	0.8641
Prescription history data (n= 355)	***Adjusted Odds Ratio***	***95% confidence interval***		***P-Value***
Victim of fraud and/or identity theft	1.266	0.812	1.975	0.2984
Age range (ref = over 60)				
18 to 30	1.323	0.674	2.594	0.0771*
31 to 40	0.922	0.495	1.717	0.7538
41 to 50	0.518	0.26	1.034	0.0473**
51 to 60	0.742	0.397	1.39	0.5149
Education (ref = Doctorate or other terminal degree)				
High school	1.298	0.327	5.147	0.8308
Some College/Associates/Trade School	1.483	0.631	3.487	0.3065
Bachelors	1.451	0.634	3.32	0.3176
Masters	0.785	0.34	1.813	0.0734*
Electronic medical record data (n= 354)	***Adjusted Odds Ratio***	***95% confidence interval***		***P-Value***
Victim of fraud and/or identity theft	1.152	0.74	1.795	0.5308
Age range (ref = over 60)				
18 to 30	0.543	0.279	1.056	0.549
31 to 40	0.705	0.378	1.317	0.5923
41 to 50	0.502	0.253	0.996	0.3802
51 to 60	0.5	0.265	0.946	0.3244
Education (ref = Doctorate or other terminal degree)				

High school	0.952	0.217	4.179	0.9654
Some College/Associates/Trade School	0.982	0.42	2.296	0.9753
Bachelors	1.21	0.534	2.745	0.3251
Masters	0.777	0.338	1.786	0.3253
**Genetic data (n= 256)**	**Adjusted Odds Ratio**	**95% confidence interval**		**P-Value**
Victim of fraud and/or identity theft	1.236	0.734	2.082	0.4251
Age range (ref = over 60)				
18 to 30	0.723	0.326	1.603	0.6707
31 to 40	0.844	0.385	1.847	0.911
41 to 50	0.633	0.298	1.345	0.358
51 to 60	0.944	0.448	1.987	0.5909
Education (ref = Doctorate or other terminal degree)				
High school	0.289	0.047	1.788	0.207
Some College/Associates/Trade School	1.077	0.402	2.885	0.1074
Bachelors	0.846	0.331	2.161	0.4006
Masters	0.536	0.206	1.398	0.4058
**Direct Communication Data**				
Text message and phone data (n= 355)	***Adjusted Odds Ratio***	***95% confidence interval***		***P-Value***
Victim of fraud and/or identity theft	1.309	0.813	2.107	0.2679
Age range (ref = over 60)				
18 to 30	1.064	0.512	2.214	0.9078
31 to 40	1.62	0.84	3.122	0.0871*
41 to 50	0.829	0.384	1.793	0.322
51 to 60	1.112	0.565	2.187	0.9562
Education (ref = Doctorate or other terminal degree)				
High school	1.109	0.257	4.789	0.6452
Some College/Associates/Trade School	1.018	0.419	2.471	0.5229
Bachelors	0.846	0.357	2.005	0.8941
Masters	0.528	0.215	1.294	0.0445**
**Email history data (n= 358)**	**Adjusted Odds Ratio**	**95% confidence interval**		**P-Value**
Victim of fraud and/or identity theft	1.334	0.835	2.131	0.2275
Age range (ref = over 60)				
18 to 30	0.798	0.388	1.642	0.4465

31 to 40	1.135	0.589	2.186	0.5047
41 to 50	0.756	0.359	1.591	0.3599
51 to 60	1.268	0.663	2.424	0.2474
Education (ref = Doctorate or other terminal degree)				
High school	2.578	0.646	10.283	0.1625
Some College/Associates/Trade School	1.717	0.719	4.103	0.2414
Bachelors	1.279	0.546	2.996	0.918
Masters	0.676	0.278	1.647	0.007**
**Online Browsing or Streaming Data**				
Music streaming data (n= 276)	***Adjusted Odds Ratio***	***95% confidence interval***		***P-Value***
Victim of fraud and/or identity theft	0.96	0.556	1.661	0.8853
Age range (ref = over 60)				
18 to 30	7.593	3.026	19.051	0.005**
31 to 40	3.48	1.601	7.563	0.6685
41 to 50	2.8	1.235	6.35	0.7088
51 to 60	3.976	1.7	9.3	0.4048
Education (ref = Doctorate or other terminal degree)				
High school	1.07	0.168	6.792	0.8949
Some College/Associates/Trade School	1.186	0.419	3.355	0.5302
Bachelors	0.743	0.279	1.98	0.3227
Masters	0.948	0.349	2.578	0.9137
**Google search history data (n= 361)**	**Adjusted Odds Ratio**	**95% confidence interval**		**P-Value**
Victim of fraud and/or identity theft	1.267	0.812	1.975	0.2969
Age range (ref = over 60)				
18 to 30	1.072	0.549	2.093	0.7873
31 to 40	1.257	0.674	2.342	0.6691
41 to 50	0.9	0.453	1.79	0.3429
51 to 60	1.612	0.859	3.026	0.1243
Education (ref = Doctorate or other terminal degree)				
High school	2.178	0.531	8.926	0.1231
Some College/Associates/Trade School	1.116	0.49	2.545	0.6138
Bachelors	0.659	0.297	1.462	0.0592*
Masters	0.601	0.267	1.351	0.0268**

**Financial Data**				
Online purchase history data (n= 359)	***Adjusted Odds Ratio***	***95% confidence interval***		***P-Value***
Victim of fraud and/or identity theft	1.256	0.807	1.954	0.3123
Age range (ref = over 60)				
18 to 30	1.611	0.83	3.126	0.5048
31 to 40	1.681	0.903	3.131	0.3598
41 to 50	1.088	0.549	2.158	0.3486
51 to 60	1.675	0.89	3.15	0.3811
Education (ref = Doctorate or other terminal degree)				
High school	3.859	0.701	21.242	0.0475**
Some College/Associates/Trade School	0.924	0.4	2.135	0.5227
Bachelors	0.72	0.321	1.615	0.0818*
Masters	0.588	0.258	1.339	0.0125**
Tax records and income history data (n= 350)	***Adjusted Odds Ratio***	***95% confidence interval***		***P-Value***
Victim of fraud and/or identity theft	1.403	0.817	2.41	0.22
Age range (ref = over 60)				
18 to 30	1.202	0.526	2.747	0.7196
31 to 40	1.747	0.85	3.592	0.0542**
41 to 50	0.934	0.39	2.235	0.6494
51 to 60	0.756	0.331	1.725	0.2264
Education (ref = Doctorate or other terminal degree)				
High school	2.635	0.46	15.1	0.1655
Some College/Associates/Trade School	0.932	0.353	2.464	0.601
Bachelors	0.729	0.283	1.882	0.1428
Masters	0.829	0.32	2.148	0.3376
Credit card statement data (n=343)	***Adjusted Odds Ratio***	***95% confidence interval***		***P-Value***
Victim of fraud and/or identity theft	1.489	0.856	2.591	0.1587
Age range (ref = over 60)				
Indent 18 to 30	0.746	0.322	1.732	0.8029
Indent 31 to 40	0.898	0.417	1.934	0.703
Indent 41 to 50	0.862	0.372	1.998	0.8327
Indent 51 to 60	0.591	0.263	1.328	0.2991
Education (ref = Doctorate or other terminal degree)				

High school	3.504	0.715	17.178	0.0498**
Some College/Associates/Trade School	1.035	0.377	2.838	0.6774
Bachelors	0.941	0.354	2.499	0.4163
Masters	0.621	0.227	1.701	0.0264**
**Location Data**				
Ridesharing history data (n= 199)	***Adjusted Odds Ratio***	***95% confidence interval***		***P-Value***
Victim of fraud and/or identity theft	1.138	0.62	2.089	0.6765
Age range (ref = over 60)				
18 to 30	3.121	1.264	7.709	0.2016
31 to 40	2.468	1.013	6.01	0.6423
41 to 50	2.432	0.934	6.333	0.7214
51 to 60	2.515	0.955	6.622	0.6427
Education (ref = Doctorate or other terminal degree)				
High school	2.183	0.274	17.369	0.5379
Some College/Associates/Trade School	1.366	0.439	4.249	0.9904
Bachelors	1.309	0.45	3.804	0.8996
Masters	1.194	0.404	3.528	0.6742
Geolocation data (n= 348)	***Adjusted Odds Ratio***	***95% confidence interval***		***P-Value***
Victim of fraud and/or identity theft	0.997	0.633	1.571	0.9895
Age range (ref = over 60)				
18 to 30	1.125	0.568	2.231	0.981
31 to 40	1.256	0.66	2.39	0.6426
41 to 50	0.891	0.437	1.818	0.352
51 to 60	1.475	0.769	2.829	0.2457
Education (ref = Doctorate or other terminal degree)				
High school	1.788	0.405	7.892	0.2669
Some College/Associates/Trade School	1.144	0.492	2.66	0.5255
Bachelors	0.843	0.372	1.913	0.4923
Masters	0.533	0.229	1.237	0.0102**
**Voting History Data (n= 348)**	***Adjusted Odds Ratio***	***95% confidence interval***		***P-Value***
Victim of fraud and/or identity theft	0.96	0.612	1.504	0.8572
Age range (ref = over 60)				
18 to 30	3.344	1.661	6.729	0.0078**

31 to 40	1.78	0.947	3.342	0.86
41 to 50	1.913	0.951	3.85	0.6642
51 to 60	1.288	0.681	2.437	0.2108
Education (ref = Doctorate or other terminal degree)				
High school	1.223	0.281	5.334	0.8401
Some College/Associates/Trade School	1.914	0.804	4.561	0.157
Bachelors	1.585	0.685	3.672	0.4976
Masters	1.259	0.541	2.928	0.7322

**Significant value ($p \leq 0.05$)

*Modestly significant value ($p \leq 0.10$)
