# Supplementary material for: A Survey of Research Participants’ Privacy-Related Experiences and Willingness to Share Real-World Data with Researchers
Source: J Pers Med. 2022 Nov 17;12(11):1922. doi: 10.3390/jpm12111922 (PMC9696408; doi:10.3390/jpm12111922)
Supplement: Supplementary file 1 [file jpm-12-01922-s001.zip › Table S8_unpleasant experience as a result of information shared online .pdf]

Table S8. Associations between willingness to share real-world data from various sources and had an unpleasant experience as a result of information posted online, adjusted for age range and education level.

**Social Media Data**				
Facebook data (n= 270)	***Adjusted Odds Ratio***	***95% confidence interval***		***P-Value***
Unpleasant experience as a result of information posted online	1.079	0.623	1.868	0.7865
Age range (ref = over 60)				
18 to 30	1.683	0.765	3.701	0.2734
31 to 40	1.054	0.502	2.214	0.5228
41 to 50	0.814	0.367	1.809	0.1401
51 to 60	2.044	0.933	4.476	0.0745*
Education (ref = Doctorate or other terminal degree)				
High school	1.823	0.278	11.941	0.4083
Some College/Associates/Trade School	1.1	0.372	3.253	0.8563
Bachelors	0.839	0.295	2.39	0.4233
Masters	0.735	0.254	2.122	0.2052
Twitter data (n= 124)	***Adjusted Odds Ratio***	***95% confidence interval***		***P-Value***
Unpleasant experience as a result of information posted online	0.729	0.304	1.752	0.48
Age range (ref = over 60)				
18 to 30	3.911	1.164	13.139	0.2153
31 to 40	2.574	0.79	8.384	0.8174
41 to 50	1.249	0.366	4.267	0.1313
51 to 60	5.728	1.443	22.735	0.0701*
Education (ref = Doctorate or other terminal degree)				
High school	0.754	0.044	13.042	0.8146
Some College/Associates/Trade School	0.302	0.05	1.812	0.1263
Bachelors	0.686	0.12	3.915	0.7414
Masters	0.491	0.083	2.898	0.6492
Instagram data (n= 189)	***Adjusted Odds Ratio***	***95% confidence interval***		***P-Value***
Unpleasant experience as a result of information posted online	1.13	0.602	2.121	0.7033
Age range (ref = over 60)				
18 to 30	1.732	0.682	4.396	0.8535
31 to 40	1.518	0.601	3.836	0.7885

41 to 50	1.875	0.687	5.118	0.6873
51 to 60	2.405	0.781	7.404	0.3244
Education (ref = Doctorate or other terminal degree)				
High school	1.947	0.16	23.643	0.598
Some College/Associates/Trade School	1.137	0.33	3.923	0.9171
Bachelors	1.332	0.41	4.331	0.7329
Masters	0.787	0.243	2.55	0.2376
Snapchat data (n= 104)	***Adjusted Odds Ratio***	***95% confidence interval***		***P-Value***
Unpleasant experience as a result of information posted online	1.51	0.644	3.538	0.3431
Age range (ref = over 60)				
18 to 30	-	-	-	-
31 to 40	-	-	-	-
41 to 50	-	-	-	-
51 to 60	-	-	-	-
Education (ref = Doctorate or other terminal degree)				
High school	0.737	0.031	17.651	0.8564
Some College/Associates/Trade School	0.224	0.037	1.353	0.0674*
Bachelors	0.543	0.106	2.787	0.8312
Masters	0.841	0.147	4.812	0.501
Yelp reviews and ratings data (n= 179)	***Adjusted Odds Ratio***	***95% confidence interval***		***P-Value***
Unpleasant experience as a result of information posted online	1.299	0.655	2.573	0.4541
Age range (ref = over 60)				
18 to 30	2.04	0.721	5.769	0.8862
31 to 40	2.196	0.84	5.74	0.716
41 to 50	2.008	0.793	5.09	0.9092
51 to 60	2.981	1.123	7.914	0.2393
Education (ref = Doctorate or other terminal degree)				
High school	-	-	-	-
Some College/Associates/Trade School	0.635	0.18	2.234	0.9693
Bachelors	0.896	0.255	3.15	0.9734
Masters	0.95	0.274	3.298	0.9741

**Health Data**				
Fitness tracker data (n= 220)	***Adjusted Odds Ratio***	***95% confidence interval***		***P-Value***
Unpleasant experience as a result of information posted online	0.906	0.459	1.788	0.7769
Age range (ref = over 60)				
18 to 30	3.385	1.228	9.328	0.2423
31 to 40	2.043	0.872	4.787	0.8466
41 to 50	1.933	0.789	4.739	0.7328
51 to 60	3.605	1.294	10.038	0.1963
Education (ref = Doctorate or other terminal degree)				
High school	0.564	0.072	4.425	0.5342
Some College/Associates/Trade School	0.997	0.331	3.009	0.7869
Bachelors	1.09	0.358	3.325	0.5968
Masters	1.012	0.352	2.907	0.7304
Prescription history data (n= 351)	***Adjusted Odds Ratio***	***95% confidence interval***		***P-Value***
Unpleasant experience as a result of information posted online	0.927	0.573	1.499	0.7561
Age range (ref = over 60)				
18 to 30	1.285	0.658	2.51	0.1139
31 to 40	0.904	0.486	1.682	0.877
41 to 50	0.5	0.248	1.009	0.0329**
51 to 60	0.872	0.468	1.626	0.9973
Education (ref = Doctorate or other terminal degree)				
High school	0.99	0.236	4.152	0.7546
Some College/Associates/Trade School	1.476	0.612	3.556	0.3062
Bachelors	1.552	0.659	3.652	0.1877
Masters	0.925	0.391	2.189	0.3149
Electronic medical record data (n= 351)	***Adjusted Odds Ratio***	***95% confidence interval***		***P-Value***
Unpleasant experience as a result of information posted online	1	0.623	1.605	0.9995
Age range (ref = over 60)				
18 to 30	0.491	0.254	0.949	0.2967
31 to 40	0.671	0.36	1.25	0.7758
41 to 50	0.518	0.259	1.035	0.4457
51 to 60	0.58	0.309	1.088	0.7194
Education (ref = Doctorate or other terminal degree)				

High school	0.68	0.138	3.35	0.5826
Some College/Associates/Trade School	0.992	0.415	2.371	0.816
Bachelors	1.205	0.518	2.8	0.2746
Masters	0.889	0.378	2.092	0.8251
**Genetic data (n= 247)**	**Adjusted Odds Ratio**	**95% confidence interval**		**P-Value**
Unpleasant experience as a result of information posted online	0.992	0.551	1.785	0.9791
Age range (ref = over 60)				
18 to 30	0.707	0.32	1.563	0.5105
31 to 40	0.851	0.386	1.88	0.9881
41 to 50	0.638	0.295	1.379	0.3047
51 to 60	1.191	0.552	2.568	0.2414
Education (ref = Doctorate or other terminal degree)				
High school	0.129	0.013	1.28	0.0879*
Some College/Associates/Trade School	1.064	0.396	2.857	0.0623*
Bachelors	0.819	0.317	2.119	0.2685
Masters	0.593	0.227	1.549	0.9514
**Direct Communication Data**				
Text message and phone data (n= 352)	***Adjusted Odds Ratio***	***95% confidence interval***		***P-Value***
Unpleasant experience as a result of information posted online	1.324	0.803	2.182	0.2709
Age range (ref = over 60)				
18 to 30	0.993	0.485	2.035	0.832
31 to 40	1.395	0.724	2.687	0.2175
41 to 50	0.716	0.321	1.599	0.1984
51 to 60	1.281	0.657	2.494	0.4045
Education (ref = Doctorate or other terminal degree)				
High school	1.474	0.324	6.712	0.4932
Some College/Associates/Trade School	1.092	0.43	2.772	0.7863
Bachelors	0.987	0.398	2.45	0.8833
Masters	0.7	0.274	1.786	0.1306
**Email history data (n= 354)**	**Adjusted Odds Ratio**	**95% confidence interval**		**P-Value**
Unpleasant experience as a result of information posted online	1.371	0.833	2.256	0.214
Age range (ref = over 60)				
18 to 30	0.765	0.375	1.56	0.3991

31 to 40	0.997	0.512	1.94	0.841
41 to 50	0.698	0.322	1.516	0.2832
51 to 60	1.457	0.759	2.796	0.0705*
Education (ref = Doctorate or other terminal degree)				
High school	3.717	0.861	16.056	0.0882*
Some College/Associates/Trade School	1.998	0.792	5.039	0.3036
Bachelors	1.573	0.633	3.907	0.9795
Masters	0.801	0.309	2.074	0.008**
**Online Browsing or Streaming Data**				
Music streaming data (n= 271)	***Adjusted Odds Ratio***	***95% confidence interval***		***P-Value***
Unpleasant experience as a result of information posted online	1.24	0.695	2.215	0.4665
Age range (ref = over 60)				
18 to 30	6.702	2.812	15.97	0.012**
31 to 40	3.737	1.73	8.071	0.4896
41 to 50	2.611	1.158	5.888	0.5397
51 to 60	4.511	1.924	10.574	0.2185
Education (ref = Doctorate or other terminal degree)				
High school	1.068	0.16	7.11	0.9828
Some College/Associates/Trade School	1.187	0.414	3.4	0.695
Bachelors	0.82	0.301	2.236	0.3744
Masters	1.24	0.441	3.485	0.5775
**Google search history data (n= 358)**	**Adjusted Odds Ratio**	**95% confidence interval**		**P-Value**
Unpleasant experience as a result of information posted online	1.305	0.814	2.094	0.2692
Age range (ref = over 60)				
18 to 30	1.041	0.537	2.02	0.7757
31 to 40	1.187	0.637	2.211	0.7784
41 to 50	0.841	0.417	1.697	0.2755
51 to 60	1.657	0.885	3.103	0.0818*
Education (ref = Doctorate or other terminal degree)				
High school	3.227	0.7	14.888	0.0633*
Some College/Associates/Trade School	1.139	0.489	2.649	0.9846
Bachelors	0.722	0.317	1.643	0.0426**
Masters	0.738	0.321	1.697	0.0597*

Financial Data				
Online purchase history data (n= 355)	***Adjusted Odds Ratio***	***95% confidence interval***		***P-Value***
Unpleasant experience as a result of information posted online	1.306	0.816	2.092	0.2663
Age range (ref = over 60)				
18 to 30	1.411	0.732	2.72	0.6974
31 to 40	1.438	0.774	2.671	0.617
41 to 50	1.01	0.503	2.028	0.3448
51 to 60	1.725	0.914	3.255	0.2052
Education (ref = Doctorate or other terminal degree)				
High school	8.378	0.927	75.706	0.0313**
Some College/Associates/Trade School	0.922	0.392	2.168	0.2001
Bachelors	0.791	0.345	1.811	0.058*
Masters	0.696	0.3	1.619	0.0217**
Tax records and income history data (n= 348)	***Adjusted Odds Ratio***	***95% confidence interval***		***P-Value***
Unpleasant experience as a result of information posted online	1.574	0.898	2.756	0.1129
Age range (ref = over 60)				
18 to 30	1.066	0.472	2.409	0.9744
31 to 40	1.586	0.768	3.277	0.1071
41 to 50	0.951	0.395	2.29	0.7455
51 to 60	0.819	0.359	1.868	0.3935
Education (ref = Doctorate or other terminal degree)				
High school	2.902	0.498	16.915	0.1431
Some College/Associates/Trade School	0.883	0.321	2.43	0.3952
Bachelors	0.769	0.287	2.06	0.1561
Masters	0.939	0.349	2.53	0.505
Credit card statement data (n=341)	***Adjusted Odds Ratio***	***95% confidence interval***		***P-Value***
Unpleasant experience as a result of information posted online	1.564	0.88	2.78	0.1273
Age range (ref = over 60)				
Indent 18 to 30	0.668	0.291	1.53	0.5828
Indent 31 to 40	0.793	0.363	1.731	0.9886
Indent 41 to 50	0.881	0.377	2.059	0.7322
Indent 51 to 60	0.659	0.294	1.479	0.5488
Education				

High school	3.52	0.7	17.706	0.0544**
Some College/Associates/Trade School	0.977	0.339	2.818	0.4811
Bachelors	0.997	0.358	2.78	0.4946
Masters	0.704	0.245	2.021	0.0618*
**Location Data**				
Ridesharing history data (n= 199)	***Adjusted Odds Ratio***	***95% confidence interval***		***P-Value***
Unpleasant experience as a result of information posted online	1.008	0.533	1.905	0.9815
Age range (ref = over 60)				
18 to 30	3.079	1.302	7.279	0.2222
31 to 40	2.173	0.898	5.257	0.9794
41 to 50	2.925	1.114	7.679	0.3922
51 to 60	2.573	0.982	6.737	0.628
Education (ref = Doctorate or other terminal degree)				
High school	2.048	0.251	16.681	0.512
Some College/Associates/Trade School	1.189	0.363	3.888	0.9032
Bachelors	1.148	0.377	3.499	0.8033
Masters	1.046	0.332	3.295	0.5914
Geolocation data (n= 342)	***Adjusted Odds Ratio***	***95% confidence interval***		***P-Value***
Unpleasant experience as a result of information posted online	1.088	0.671	1.763	0.7318
Age range (ref = over 60)				
18 to 30	1.179	0.6	2.317	0.8837
31 to 40	1.392	0.733	2.642	0.563
41 to 50	0.981	0.478	2.013	0.3987
51 to 60	1.689	0.879	3.245	0.1643
Education (ref = Doctorate or other terminal degree)				
High school	1.459	0.313	6.792	0.4472
Some College/Associates/Trade School	1.021	0.429	2.431	0.7827
Bachelors	0.887	0.381	2.069	0.748
Masters	0.601	0.253	1.431	0.055*
**Voting History Data (n= 344)**	***Adjusted Odds Ratio***	***95% confidence interval***		***P-Value***
Unpleasant experience as a result of information posted online	1.002	0.618	1.624	0.9932
Age range (ref = over 60)				
18 to 30	3.023	1.533	5.959	0.026**

31 to 40	1.86	0.993	3.484	0.7862
41 to 50	1.999	0.981	4.076	0.6127
51 to 60	1.462	0.771	2.772	0.4383
Education (ref = Doctorate or other terminal degree)				
High school	0.999	0.213	4.676	0.6595
Some College/Associates/Trade School	1.755	0.72	4.278	0.1941
Bachelors	1.553	0.654	3.691	0.3931
Masters	1.245	0.52	2.98	0.9157

**Significant value (p≤ 0.05)

*Modestly significant value (p≤ 0.10)
