# Supplementary material for: A Survey of Research Participants’ Privacy-Related Experiences and Willingness to Share Real-World Data with Researchers
Source: J Pers Med. 2022 Nov 17;12(11):1922. doi: 10.3390/jpm12111922 (PMC9696408; doi:10.3390/jpm12111922)
Supplement: Supplementary file 1 [file jpm-12-01922-s001.zip › Table S9_Reputation Neg Affected.pdf]

Table S9. Associations between willingness to share real-world data from various sources and experienced reputation being negatively affected as a result of information posted online, adjusted for age range and education level.

**Real-World Data Source**	**Logistic regression results**			
**Social Media Data**				
Facebook data (n= 248)	***Adjusted Odds Ratio***	***95% confidence interval***		***P-Value***
Reputation being negatively affected as a result of information posted online	1.78	0.71	4.466	0.2189
Age range (ref = over 60)				
18 to 30	1.431	0.622	3.294	0.1663
31 to 40	0.771	0.351	1.69	0.4037
41 to 50	0.555	0.239	1.288	0.0613*
51 to 60	1.339	0.593	3.022	0.2361
Education (ref = Doctorate or other terminal degree)				
High school	1.873	0.281	12.498	0.5157
Some College/Associates/Trade School	1.418	0.501	4.015	0.5666
Bachelors	1.128	0.418	3.046	0.8507
Masters	0.794	0.29	2.171	0.157
Twitter data (n= 117)	***Adjusted Odds Ratio***	***95% confidence interval***		***P-Value***
Reputation being negatively affected as a result of information posted online	3.278	0.608	17.678	0.1673
Age range (ref = over 60)				
18 to 30	3.053	0.844	11.045	0.2482
31 to 40	1.601	0.466	5.502	0.7336
41 to 50	1.06	0.295	3.803	0.1924
51 to 60	4.143	1.021	16.822	0.1015*
Education (ref = Doctorate or other terminal degree)				
High school	0.854	0.055	13.275	0.8397
Some College/Associates/Trade School	0.396	0.078	2.008	0.22
Bachelors	0.755	0.16	3.569	0.8502
Masters	0.649	0.132	3.185	0.8704
Instagram data (n= 177)	***Adjusted Odds Ratio***	***95% confidence interval***		***P-Value***
Reputation being negatively affected as a result of information posted online	1.604	0.54	4.768	0.3952
Age range (ref = over 60)				
18 to 30	1.345	0.5	3.618	0.7519

31 to 40	1.132	0.42	3.053	0.7912
41 to 50	1.301	0.446	3.795	0.8554
51 to 60	1.385	0.433	4.432	0.7469
Education (ref = Doctorate or other terminal degree)				
High school	2.25	0.187	27.133	0.6315
Some College/Associates/Trade School	1.444	0.424	4.912	0.9789
Bachelors	1.742	0.546	5.555	0.5743
Masters	1.053	0.33	3.366	0.3912
Snapchat data (n= 99)	***Adjusted Odds Ratio***	***95% confidence interval***		***P-Value***
Reputation being negatively affected as a result of information posted online	1.68	0.476	5.929	0.4198
Age range (ref = over 60)				
18 to 30	-	-	-	-
31 to 40	-	-	-	-
41 to 50	-	-	-	-
51 to 60	-	-	-	-
Education (ref = Doctorate or other terminal degree)				
High school	0.86	0.035	21.393	0.9835
Some College/Associates/Trade School	0.369	0.064	2.112	0.1295
Bachelors	0.798	0.166	3.822	0.9075
Masters	1.645	0.297	9.112	0.2119
Yelp reviews and ratings data (n= 160)	***Adjusted Odds Ratio***	***95% confidence interval***		***P-Value***
Reputation being negatively affected as a result of information posted online	2.081	0.704	6.151	0.1851
Age range (ref = over 60)				
18 to 30	1.781	0.604	5.247	0.971
31 to 40	2.14	0.792	5.786	0.6357
41 to 50	1.882	0.714	4.962	0.9066
51 to 60	2.681	0.956	7.521	0.297
Education (ref = Doctorate or other terminal degree)				
High school	-	-	-	-
Some College/Associates/Trade School	0.959	0.278	3.308	0.9706
Bachelors	1.145	0.338	3.885	0.9731
Masters	1.167	0.348	3.909	0.9734
**Health Data**				

Fitness tracker data (n= 207)	*Adjusted Odds Ratio*	*95% confidence interval*		*P-Value*
Reputation being negatively affected as a result of information posted online	1.909	0.517	7.053	0.332
Age range (ref = over 60)				
18 to 30	2.012	0.671	6.029	0.4215
31 to 40	1.421	0.559	3.611	0.9457
41 to 50	1.059	0.419	2.676	0.3444
51 to 60	2.142	0.728	6.304	0.3344
Education (ref = Doctorate or other terminal degree)				
High school	0.53	0.068	4.126	0.4616
Some College/Associates/Trade School	0.887	0.292	2.693	0.8889
Bachelors	1.507	0.48	4.734	0.196
Masters	0.997	0.354	2.812	0.8354
Prescription history data (n= 329)	*Adjusted Odds Ratio*	*95% confidence interval*		*P-Value*
Reputation being negatively affected as a result of information posted online	1.621	0.757	3.468	0.2134
Age range (ref = over 60)				
18 to 30	1.173	0.591	2.329	0.1223
31 to 40	0.886	0.466	1.683	0.6659
41 to 50	0.436	0.21	0.903	0.0213**
51 to 60	0.738	0.385	1.413	0.7135
Education (ref = Doctorate or other terminal degree)				
High school	1.164	0.266	5.099	0.9505
Some College/Associates/Trade School	1.343	0.563	3.2	0.472
Bachelors	1.369	0.592	3.168	0.3893
Masters	0.846	0.363	1.972	0.2205
Electronic medical record data (n= 326)	*Adjusted Odds Ratio*	*95% confidence interval*		*P-Value*
Reputation being negatively affected as a result of information posted online	2.074	0.986	4.364	0.0546*
Age range (ref = over 60)				
18 to 30	0.418	0.211	0.829	0.3191
31 to 40	0.562	0.291	1.084	0.8091
41 to 50	0.44	0.213	0.906	0.4675
51 to 60	0.411	0.21	0.802	0.2754
Education (ref = Doctorate or other terminal degree)				
High school	0.62	0.12	3.192	0.6885

Some College/Associates/Trade School	0.703	0.292	1.691	0.6515
Bachelors	1.023	0.44	2.377	0.2763
Masters	0.689	0.293	1.619	0.58
Genetic data (n= 234)	***Adjusted Odds Ratio***	***95% confidence interval***		***P-Value***
Reputation being negatively affected as a result of information posted online	2.302	0.894	5.93	0.0842*
Age range (ref = over 60)				
18 to 30	0.495	0.218	1.123	0.2915
31 to 40	0.611	0.265	1.406	0.7405
41 to 50	0.455	0.204	1.016	0.1779
51 to 60	1.018	0.46	2.25	0.1521
Education (ref = Doctorate or other terminal degree)				
High school	0.158	0.015	1.639	0.13
Some College/Associates/Trade School	1.061	0.386	2.923	0.1055
Bachelors	0.86	0.329	2.245	0.2839
Masters	0.616	0.231	1.642	0.9981
**Direct Communication Data**				
Text message and phone data (n= 325)	***Adjusted Odds Ratio***	***95% confidence interval***		***P-Value***
Reputation being negatively affected as a result of information posted online	1.475	0.706	3.078	0.3009
Age range (ref = over 60)				
18 to 30	1.049	0.5	2.2	0.9897
31 to 40	1.34	0.672	2.674	0.2962
41 to 50	0.765	0.334	1.748	0.2977
51 to 60	1.16	0.574	2.346	0.6696
Education (ref = Doctorate or other terminal degree)				
High school	1.469	0.314	6.865	0.3975
Some College/Associates/Trade School	1	0.402	2.484	0.7437
Bachelors	0.825	0.339	2.01	0.6509
Masters	0.544	0.216	1.37	0.0451**
Email history data (n= 328)	***Adjusted Odds Ratio***	***95% confidence interval***		***P-Value***
Reputation being negatively affected as a result of information posted online	1.705	0.821	3.539	0.1522
Age range (ref = over 60)				
18 to 30	0.691	0.33	1.448	0.337
31 to 40	0.918	0.46	1.831	0.9033

41 to 50	0.692	0.312	1.532	0.3844
51 to 60	1.28	0.649	2.524	0.132
Education (ref = Doctorate or other terminal degree)				
High school	4.022	0.893	18.118	0.0614*
Some College/Associates/Trade School	1.827	0.742	4.502	0.3861
Bachelors	1.302	0.536	3.163	0.5965
Masters	0.732	0.29	1.846	0.007**
**Online Browsing or Streaming Data**				
Music streaming data (n= 253)	***Adjusted Odds Ratio***	***95% confidence interval***		***P-Value***
Reputation being negatively affected as a result of information posted online	2.851	0.997	8.147	0.0506**
Age range (ref = over 60)				
18 to 30	6.48	2.607	16.102	0.006**
31 to 40	3.335	1.487	7.48	0.4644
41 to 50	2.149	0.921	5.015	0.4002
51 to 60	3.338	1.377	8.091	0.5132
Education (ref = Doctorate or other terminal degree)				
High school	1.104	0.166	7.355	0.9821
Some College/Associates/Trade School	1.232	0.437	3.469	0.6925
Bachelors	0.95	0.357	2.526	0.6376
Masters	1.174	0.43	3.209	0.7978
Google search history data (n= 331)	***Adjusted Odds Ratio***	***95% confidence interval***		***P-Value***
Reputation being negatively affected as a result of information posted online	1.413	0.684	2.918	0.3507
Age range (ref = over 60)				
18 to 30	0.961	0.484	1.907	0.6506
31 to 40	1.173	0.612	2.248	0.6949
41 to 50	0.841	0.405	1.744	0.3572
51 to 60	1.499	0.78	2.879	0.1464
Education (ref = Doctorate or other terminal degree)				
High school	2.587	0.554	12.079	0.1*
Some College/Associates/Trade School	1.039	0.449	2.407	0.9323
Bachelors	0.64	0.283	1.448	0.0465**
Masters	0.634	0.278	1.449	0.049**

**Financial Data**				
Online purchase history data (n= 328)	***Adjusted Odds Ratio***	***95% confidence interval***		***P-Value***
Reputation being negatively affected as a result of information posted online	1.417	0.681	2.951	0.3515
Age range (ref = over 60)				
18 to 30	1.318	0.67	2.592	0.8661
31 to 40	1.445	0.755	2.764	0.5601
41 to 50	1.101	0.534	2.267	0.5915
51 to 60	1.552	0.802	3.005	0.3814
Education (ref = Doctorate or other terminal degree)				
High school	6.775	0.746	61.563	0.0446**
Some College/Associates/Trade School	0.882	0.377	2.064	0.2814
Bachelors	0.72	0.317	1.638	0.0636*
Masters	0.607	0.263	1.402	0.017**
Tax records and income history data (n= 322)	***Adjusted Odds Ratio***	***95% confidence interval***		***P-Value***
Reputation being negatively affected as a result of information posted online	1.256	0.546	2.89	0.5921
Age range (ref = over 60)				
18 to 30	1.143	0.493	2.648	0.86
31 to 40	1.749	0.822	3.725	0.0615*
41 to 50	0.959	0.378	2.435	0.7148
51 to 60	0.786	0.328	1.882	0.2955
Education (ref = Doctorate or other terminal degree)				
High school	2.776	0.441	17.463	0.1404
Some College/Associates/Trade School	0.705	0.26	1.911	0.235
Bachelors	0.78	0.302	2.012	0.3382
Masters	0.709	0.27	1.867	0.2246
Credit card statement data (n=315)	***Adjusted Odds Ratio***	***95% confidence interval***		***P-Value***
Reputation being negatively affected as a result of information posted online	1.457	0.623	3.405	0.3854
Age range (ref = over 60)				
Indent 18 to 30	0.714	0.305	1.676	0.6851
Indent 31 to 40	0.934	0.423	2.061	0.6165
Indent 41 to 50	0.861	0.353	2.098	0.8528
Indent 51 to 60	0.607	0.258	1.424	0.354
Education (ref = Doctorate or other terminal degree)				

High school	3.52	0.68	18.221	0.0499**
Some College/Associates/Trade School	0.886	0.316	2.48	0.4342
Bachelors	0.985	0.369	2.632	0.6406
Masters	0.563	0.202	1.573	0.0219**
**Location Data**				
Ridesharing history data (n= 182)	***Adjusted Odds Ratio***	***95% confidence interval***		***P-Value***
Reputation being negatively affected as a result of information posted online	1.722	0.604	4.915	0.3095
Age range (ref = over 60)				
18 to 30	2.296	0.919	5.738	0.3939
31 to 40	2.063	0.819	5.2	0.6394
41 to 50	1.914	0.699	5.241	0.8492
51 to 60	2.048	0.753	5.572	0.6921
Education (ref = Doctorate or other terminal degree)				
High school	1.994	0.255	15.616	0.5974
Some College/Associates/Trade School	1.23	0.391	3.868	0.8189
Bachelors	1.429	0.489	4.177	0.8254
Masters	1.201	0.406	3.557	0.7437
Geolocation data (n= 320)	***Adjusted Odds Ratio***	***95% confidence interval***		***P-Value***
Reputation being negatively affected as a result of information posted online	1.278	0.611	2.672	0.5147
Age range (ref = over 60)				
18 to 30	0.993	0.497	1.983	0.8738
31 to 40	1.213	0.625	2.355	0.4831
41 to 50	0.82	0.39	1.726	0.388
51 to 60	1.186	0.602	2.338	0.5595
Education (ref = Doctorate or other terminal degree)				
High school	1.134	0.232	5.542	0.6235
Some College/Associates/Trade School	1.012	0.428	2.39	0.4915
Bachelors	0.771	0.334	1.777	0.6731
Masters	0.506	0.215	1.194	0.0382**
Voting History Data (n= 318)	***Adjusted Odds Ratio***	***95% confidence interval***		***P-Value***
Reputation being negatively affected as a result of information posted online	1.175	0.552	2.501	0.6747
Age range (ref = over 60)				
18 to 30	2.835	1.394	5.766	0.0065**

31 to 40	1.447	0.752	2.786	0.9307
41 to 50	1.421	0.683	2.955	0.9955
51 to 60	0.985	0.505	1.925	0.1261
Education (ref = Doctorate or other terminal degree)				
High school	1.346	0.268	6.761	0.9197
Some College/Associates/Trade School	1.964	0.805	4.789	0.214
Bachelors	1.88	0.792	4.462	0.2516
Masters	1.197	0.503	2.85	0.4704

**Significant value (p≤ 0.05)

*Modestly significant value (p≤ 0.10)
