# Supplementary material for: A Survey of Research Participants’ Privacy-Related Experiences and Willingness to Share Real-World Data with Researchers
Source: J Pers Med. 2022 Nov 17;12(11):1922. doi: 10.3390/jpm12111922 (PMC9696408; doi:10.3390/jpm12111922)
Supplement: Supplementary file 1 [file jpm-12-01922-s001.zip › Tables S2-S6 - Participants willingness to donate data sources_privacy-related experiences.pdf]

Table S2. Participants' willingness to donate data sources who have been victims of fraud and/or identity theft online.

Victim of fraud and/or identity theft online	Yes				No				Unsure			
**Data Source**	Willingness to share data sources with researchers				Willingness to share data sources with researchers				Willingness to share data sources with researchers			
	Yes	No	I don't use this source of data	Total (n)	Yes	No	I don't use this source of data	Total (n)	Yes	No	I don't use this source of data	Total (n)
**Facebook data**	44%	29%	27%	158	45%	31%	25%	204	53%	26%	21%	34
**Twitter data**	22%	18%	60%	156	22%	11%	67%	204	21%	12%	68%	34
**Instagram data**	34%	23%	44%	155	31%	21%	49%	204	26%	18%	56%	34
**Snapchat data**	11%	20%	69%	157	13%	16%	71%	204	6%	21%	74%	34
**Email History (Gmail, Yahoo, Comcast, Verizon, etc.)**	36%	61%	3%	157	30%	70%	0%	203	32%	68%	0%	34
**Text Message and Phone Call Data**	30%	66%	3%	158	29%	69%	2%	204	29%	71%	0%	34
**Google search history**	43%	57%	0%	157	42%	57%	1%	204	44%	56%	0%	34
**Online purchase history (Amazon, Target, Ebay, etc.)**	43%	55%	1%	157	49%	50%	1%	204	41%	59%	0%	34
**Music streaming data (Spotify, Pandora, etc.)**	48%	30%	22%	158	50%	23%	27%	204	41%	26%	32%	34
**Yelp reviews and ratings**	37%	17%	46%	156	28%	18%	54%	204	24%	26%	50%	34
**Ride-sharing history (Uber, Lyft, etc.)**	24%	31%	46%	157	27%	27%	45%	204	12%	29%	59%	34

**Fitness tracker data (FitBit, Apple Watch, etc.)**	48%	16%	35%	158	43%	16%	42%	204	44%	26%	29%	34
**Tax records and income history**	19%	76%	4%	157	20%	76%	3%	204	12%	88%	0%	34
**Credit card statement data**	19%	75%	6%	157	19%	75%	6%	204	12%	85%	3%	34
**Voting history**	44%	52%	4%	155	51%	45%	4%	203	35%	65%	0%	34
**Prescription history (CVS, Walgreen's, etc.)**	50%	48%	2%	157	53%	44%	3%	204	35%	65%	0%	34
**Electronic medical record data**	45%	54%	1%	157	46%	51%	2%	202	38%	62%	0%	34
**Geolocation (GPS from your phone or computer) data**	40%	56%	4%	158	37%	58%	5%	203	35%	62%	3%	34
**Genetic data (23andMe, etc.)**	36%	35%	28%	158	33%	35%	32%	203	24%	38%	38%	34

Table S3. Participants' willingness to donate data sources who have had an unpleasant experience as a result of information given out online.

**Had an unpleasant experience as a result of information given out online**	**Yes**				**No**				**Unsure**			
**Data Source**	Willingness to share data sources with researchers				Willingness to share data sources with researchers				Willingness to share data sources with researchers			
	Yes	No	I don't use this source of data	Total (n)	Yes	No	I don't use this source of data	Total (n)	Yes	No	I don't use this source of data	Total (n)
**Facebook data**	50%	28%	22%	108	43%	32%	26%	250	47%	24%	29%	38
**Twitter data**	20%	17%	64%	107	22%	13%	65%	249	24%	13%	63%	38
**Instagram data**	40%	22%	38%	108	29%	21%	50%	248	22%	22%	57%	37
**Snapchat data**	13%	24%	63%	107	12%	16%	73%	250	8%	18%	74%	38
**Email History (Gmail, Yahoo, Comcast, Verizon, etc.)**	38%	61%	1%	108	31%	69%	1%	248	29%	66%	5%	38
**Text Message and Phone Call Data**	31%	69%	1%	108	29%	68%	3%	250	29%	71%	0%	38
**Google search history**	45%	54%	1%	108	42%	57%	0%	249	37%	63%	0%	38
**Online purchase history (Amazon, Target, Ebay, etc.)**	49%	50%	1%	108	45%	53%	2%	249	45%	55%	0%	38
**Music streaming data (Spotify, Pandora, etc.)**	53%	24%	23%	108	47%	27%	26%	250	47%	26%	26%	38
**Yelp reviews and ratings**	35%	18%	47%	108	31%	17%	52%	249	24%	27%	49%	37
**Ride-sharing history (Uber, Lyft, etc.)**	26%	30%	44%	108	25%	30%	45%	249	18%	21%	61%	38

**Fitness tracker data (FitBit, Apple Watch, etc.)**	50%	14%	36%	108	42%	17%	40%	250	47%	24%	29%	38
**Tax records and income history**	19%	78%	3%	108	20%	77%	4%	249	13%	82%	5%	38
**Credit card statement data**	19%	75%	6%	108	20%	76%	5%	249	11%	79%	11%	38
**Voting history**	48%	48%	4%	108	48%	49%	4%	246	39%	58%	3%	38
**Prescription history (CVS, Walgreen's, etc.)**	52%	45%	3%	108	51%	47%	2%	249	42%	58%	0%	38
**Electronic medical record data**	45%	54%	1%	108	45%	53%	2%	247	42%	58%	0%	38
**Geolocation (GPS from your phone or computer) data**	43%	55%	3%	108	36%	58%	6%	249	39%	58%	3%	38
**Genetic data (23andMe, etc.)**	33%	38%	29%	108	34%	34%	32%	249	32%	37%	32%	38

Table S4. Participants' willingness to donate data sources whose reputation was negatively affected as a result of information posted online.

Reputation was negatively affected as a result of information posted online	Yes				No				Unsure			
**Data Source**	Willingness to share data sources with researchers				Willingness to share data sources with researchers				Willingness to share data sources with researchers			
	Yes	No	I don't use this source of data	Total (n)	Yes	No	I don't use this source of data	Total (n)	Yes	No	I don't use this source of data	Total (n)
**Facebook data**	54%	23%	23%	35	44%	30%	26%	296	47%	31%	22%	64
**Twitter data**	26%	6%	68%	34	21%	13%	67%	296	24%	24%	52%	63
**Instagram data**	34%	11%	54%	35	30%	22%	48%	293	34%	25%	41%	64
**Snapchat data**	11%	11%	77%	35	12%	17%	71%	296	11%	29%	60%	63
**Email History (Gmail, Yahoo, Comcast, Verizon, etc.)**	43%	57%	0%	35	32%	67%	1%	294	31%	67%	2%	64
**Text Message and Phone Call Data**	29%	71%	0%	35	29%	68%	3%	296	31%	67%	2%	64
**Google search history**	49%	51%	0%	35	42%	58%	1%	295	44%	56%	0%	64
**Online purchase history (Amazon, Target, Ebay, etc.)**	63%	37%	0%	35	46%	53%	2%	296	40%	60%	0%	63
**Music streaming data (Spotify, Pandora, etc.)**	51%	14%	34%	35	48%	28%	25%	296	52%	25%	23%	64
**Yelp reviews and ratings**	31%	9%	60%	35	30%	19%	51%	294	36%	19%	45%	64
**Ride-sharing history (Uber, Lyft, etc.)**	26%	17%	57%	35	26%	30%	43%	295	14%	30%	56%	64

**Fitness tracker data (FitBit, Apple Watch, etc.)**	51%	3%	46%	35	43%	18%	40%	296	50%	22%	28%	64
**Tax records and income history**	20%	77%	3%	35	20%	77%	4%	295	16%	81%	3%	64
**Credit card statement data**	23%	74%	3%	35	19%	75%	6%	295	14%	81%	5%	64
**Voting history**	43%	51%	6%	35	49%	47%	4%	293	40%	59%	2%	63
**Prescription history (CVS, Walgreen's, etc.)**	63%	34%	3%	35	50%	48%	2%	295	45%	52%	3%	64
**Electronic medical record data**	60%	40%	0%	35	44%	54%	2%	293	41%	59%	0%	64
**Geolocation (GPS from your phone or computer) data**	51%	46%	3%	35	38%	57%	5%	295	33%	63%	5%	64
**Genetic data (23andMe, etc.)**	46%	20%	34%	35	32%	37%	32%	295	36%	38%	27%	64

Table S5. Participants' willingness to donate data sources whose privacy of personal information was violated.

Privacy of personal information was violated	Yes				No				Unsure			
**Data Source**	Willingness to share data sources with researchers				Willingness to share data sources with researchers				Willingness to share data sources with researchers			
	Yes	No	I don't use this source of data	Total (n)	Yes	No	I don't use this source of data	Total (n)	Yes	No	I don't use this source of data	Total (n)
**Facebook data**	42%	31%	27%	194	48%	29%	23%	131	49%	27%	24%	74
**Twitter data**	21%	15%	64%	194	24%	16%	60%	129	20%	7%	73%	74
**Instagram data**	31%	24%	45%	194	33%	19%	48%	129	38%	13%	49%	53
**Snapchat data**	12%	19%	68%	193	12%	20%	68%	131	13%	13%	75%	55
**Email History (Gmail, Yahoo, Comcast, Verizon, etc.)**	31%	68%	1%	194	33%	64%	3%	130	35%	65%	0%	54
**Text Message and Phone Call Data**	30%	69%	2%	194	30%	66%	4%	131	31%	67%	2%	55
**Google search history**	43%	56%	1%	193	44%	56%	0%	131	41%	57%	2%	54
**Online purchase history (Amazon, Target, Ebay, etc.)**	43%	55%	2%	194	49%	50%	1%	130	49%	51%	0%	74
**Music streaming data (Spotify, Pandora, etc.)**	47%	30%	23%	194	49%	24%	27%	131	51%	20%	28%	74
**Yelp reviews and ratings**	35%	22%	43%	193	30%	15%	56%	131	26%	14%	60%	73
**Ride-sharing history (Uber, Lyft, etc.)**	22%	30%	48%	194	28%	31%	41%	130	24%	23%	53%	74

**Fitness tracker data (FitBit, Apple Watch, etc.)**	48%	20%	31%	194	44%	16%	40%	131	39%	9%	51%	74
**Tax records and income history**	21%	77%	2%	194	18%	78%	5%	130	19%	76%	5%	74
**Credit card statement data**	17%	77%	6%	193	19%	76%	5%	131	23%	70%	7%	74
**Voting history**	46%	50%	4%	193	46%	51%	3%	128	51%	46%	3%	74
**Prescription history (CVS, Walgreen's, etc.)**	47%	51%	2%	193	53%	44%	2%	131	54%	43%	3%	74
**Electronic medical record data**	43%	56%	1%	193	45%	51%	4%	130	49%	49%	1%	73
**Geolocation (GPS from your phone or computer) data**	38%	59%	3%	194	39%	56%	5%	130	39%	53%	8%	74
**Genetic data (23andMe, etc.)**	32%	35%	34%	194	38%	37%	25%	130	32%	34%	34%	74

Table S6. Participants' willingness to donate data sources whose account accessed by someone without permission.

**Account accessed by someone without permission**	**Yes**				**No**				**Unsure**			
**Data Source**	Willingness to share data sources with researchers				Willingness to share data sources with researchers				Willingness to share data sources with researchers			
	Yes	No	I don't use this source of data	Total (n)	Yes	No	I don't use this source of data	Total (n)	Yes	No	I don't use this source of data	Total (n)
**Facebook data**	46%	27%	27%	193	46%	32%	22%	128	45%	31%	24%	55
**Twitter data**	26%	11%	63%	192	17%	15%	68%	127	22%	18%	60%	55
**Instagram data**	35%	21%	44%	191	29%	23%	48%	128	30%	19%	52%	54
**Snapchat data**	13%	16%	71%	192	12%	20%	69%	128	11%	22%	67%	55
**Email History (Gmail, Yahoo, Comcast, Verizon, etc.)**	33%	65%	2%	192	32%	67%	1%	128	31%	67%	2%	54
**Text Message and Phone Call Data**	32%	65%	3%	193	28%	70%	2%	128	29%	69%	2%	55
**Google search history**	42%	58%	0%	192	44%	55%	1%	128	45%	53%	2%	55
**Online purchase history (Amazon, Target, Ebay, etc.)**	45%	53%	2%	192	46%	53%	1%	128	56%	44%	0%	55
**Music streaming data (Spotify, Pandora, etc.)**	49%	30%	22%	193	47%	23%	30%	128	51%	22%	27%	55
**Yelp reviews and ratings**	30%	21%	48%	192	29%	16%	55%	127	40%	11%	49%	55
**Ride-sharing history (Uber, Lyft, etc.)**	25%	31%	44%	192	25%	28%	47%	128	25%	24%	51%	55

**Fitness tracker data (FitBit, Apple Watch, etc.)**	45%	15%	40%	193	45%	19%	36%	128	47%	20%	33%	55
**Tax records and income history**	22%	75%	3%	192	17%	77%	5%	128	15%	84%	2%	55
**Credit card statement data**	20%	73%	7%	192	19%	77%	4%	128	15%	78%	7%	55
**Voting history**	47%	49%	4%	190	51%	46%	3%	127	38%	58%	4%	55
**Prescription history (CVS, Walgreen's, etc.)**	51%	46%	3%	192	50%	48%	2%	128	53%	44%	4%	55
**Electronic medical record data**	47%	51%	2%	192	44%	55%	2%	126	42%	56%	2%	55
**Geolocation (GPS from your phone or computer) data**	42%	54%	4%	193	30%	65%	6%	127	42%	51%	7%	55
**Genetic data (23andMe, etc.)**	36%	35%	29%	193	31%	39%	29%	127	33%	29%	38%	55
